# A Novel Pore-Forming Toxin in Type A *Clostridium perfringens* Is Associated with Both Fatal Canine Hemorrhagic Gastroenteritis and Fatal Foal Necrotizing Enterocolitis

**DOI:** 10.1371/journal.pone.0122684

**Published:** 2015-04-08

**Authors:** Iman Mehdizadeh Gohari, Valeria R. Parreira, Victoria J. Nowell, Vivian M. Nicholson, Kaitlyn Oliphant, John F. Prescott

**Affiliations:** Department of Pathobiology, University of Guelph, Guelph, Ontario, Canada; Oregon State University, UNITED STATES

## Abstract

A role for type A *Clostridium perfringens* in acute hemorrhagic and necrotizing gastroenteritis in dogs and in necrotizing enterocolitis of neonatal foals has long been suspected but incompletely characterized. The supernatants of an isolate made from a dog and from a foal that died from these diseases were both found to be highly cytotoxic for an equine ovarian (EO) cell line. Partial genome sequencing of the canine isolate revealed three novel putative toxin genes encoding proteins related to the pore-forming Leukocidin/Hemolysin Superfamily; these were designated *netE*, *netF*, and *netG*. *netE* and *netF* were located on one large conjugative plasmid, and *netG* was located with a *cpe* enterotoxin gene on a second large conjugative plasmid. Mutation and complementation showed that only netF was associated with the cytotoxicity. Although *netE* and *netG* were not associated with cytotoxicity, immunoblotting with specific antisera showed these proteins to be expressed *in vitro*. There was a highly significant association between the presence of *netF* with type A strains isolated from cases of canine acute hemorrhagic gastroenteritis and foal necrotizing enterocolitis. *netE* and *netF* were found in all cytotoxic isolates, as was *cpe*, but *netG* was less consistently present. Pulsed-field gel electrophoresis showed that *netF*-positive isolates belonged to a clonal population; some canine and equine *netF*-positive isolates were genetically indistinguishable. Equine antisera to recombinant Net proteins showed that only antiserum to rNetF had high supernatant cytotoxin neutralizing activity. The identifica-tion of this novel necrotizing toxin is an important advance in understanding the virulence of type A *C*. *perfringens* in specific enteric disease of animals.

## Introduction

*C*. *perfringens* is an important Gram-positive anaerobic pathogen of humans and animals that is found ubiquitously in soil and the gastrointestinal tract of vertebrates. It causes a number of histotoxic infections, enteritis and enterotoxemias. The species produces an array of extracellular toxins, four of which (alpha, beta, epsilon and iota) form the basis for a toxin-typing scheme, which identifies five toxin types (types A, B, C, D or E) [1]. In recent years, a novel toxin, NetB, was shown to be produced by the majority of type A isolates recovered from chickens with necrotic enteritis (NE), an important disease in broiler chicken production, and to play a critical role in NE pathogenesis [2]. This important advance raises the possibility that type A strains in a number of other poorly understood but clinically and pathologically distinct enteric diseases of different animal species [1] might contain other as-yet-undescribed necrotizing toxin genes [3].

A number of important toxins, *C*. *perfringens* enterotoxin (CPE, in non-food-poisoning strains and in a minority of food poisoning strains), and all the typing toxins except for alpha-toxin (CPA), are encoded on a conserved family of large plasmids related to the pCW3 tetracycline-resistance plasmid. These plasmids share a conserved core region that includes the transfer of clostridial plasmid (*tcp*) locus required for conjugation [4,5]. The transmissible nature of key *C*. *perfringens* toxin and related virulence genes suggests that virulence of different toxin types can change through plasmid acquisition or loss. However, phylogenetic studies of *C*. *perfringens* disease strains also suggest a contribution of the chromosomal background to virulence that varies with the source of the strain. For example, clonality has been described for the majority of bovine type E isolates, for porcine type C isolates, and for isolates from chickens with NE [6–9].

*C*. *perfringens* type A-associated diarrhea and enteric disease in dogs is not well characterized, but its association with disease may range in severity from mild and self-limiting to fatal acute hemorrhagic diarrhea [10]. The acute hemorrhagic gastroenteritis form of disease is marked by severe necrotizing inflammation of the intestinal tract, especially of the small intestine, by hemorrhage and in some cases by rapid death [11,12]. The presence of large numbers of *C*. *perfringens* adhering to the necrotic intestinal mucosa is a striking and common feature [11–14]. Morbidity may be more common than mortality. Because the infection is not well characterized, no gold standard for diagnosis exists [10]. An association of *cpe*-positive *C*. *perfringens* with a case of fatal canine hemorrhagic enteritis has been described [13]. Although not well characterized, acute hemorrhagic gastroenteritis associated with *C*. *perfringens* occurs particularly in small breed dogs [15].

The role of type A *C*. *perfringens* in enteric diseases of horses is also not well understood. There is evidence that CPB2 toxin-producing *C*. *perfringens* play a role in the fatal progression of colitis in horses [16,17]. Vilei and others [18] demonstrated that some out-of-frame *cpb2*-positive equine disease isolates produced the CPB2 toxin when grown in sub-inhibitory concentrations of gentamicin. They proposed a feasible direct role of these isolates in antibiotic-associated diarrhea in horses, since treatment with aminoglycoside antibiotics allowed translation of the *cpb2* mRNA through induction of a ribosomal frame-shift. Anecdotally, there was an association between the isolation of *cpb2*-positive *C*. *perfringens* from cases of equine colitis and the use of gentamicin in hospitalized diarrheic horses, which ended when the antibiotic was stopped [18]. The correlation between CPB2 with severe and sometimes fatal colitis in horses is intriguing but the association remains unproven [19]. An apparent association has also been noted between the presence of the *C*. *perfringens* enterotoxin (CPE) and diarrheal illness in adult horses and in foals, including severe enteric disease [20–23]. Type A *C*. *perfringens* with *cbp2* and (rarely) *cpe* genes are commonly found in the feces of healthy foals, whereas type C is seldom found in healthy horses [24]. Considerable work has been done on the important role of type C *C*. *perfringens* in foal neonatal enterocolitis [25,26]. The role of type A in fatal enterocolitis in foals is less clear, but necrosis of the small intestine and colon in 1-14-day-old foals caused by type A *C*. *perfringens* has been described in Kentucky [27]. These isolates possessed both *cpb2* and *cpe* [28]. Other sporadic case reports in neonatal foals associate type A *C*. *perfringens* with fatal enterocolitis [29,30].

The present study describes a novel pore-forming toxin toxigenic *C*. *perfringens* associated with fatal hemorrhagic gastroenteritis of dogs and fatal necrotizing enterocolitis of neonatal foals.

## Results

### Identification and Analysis of Novel Putative Necrotizing Toxins

**Fig 1:**
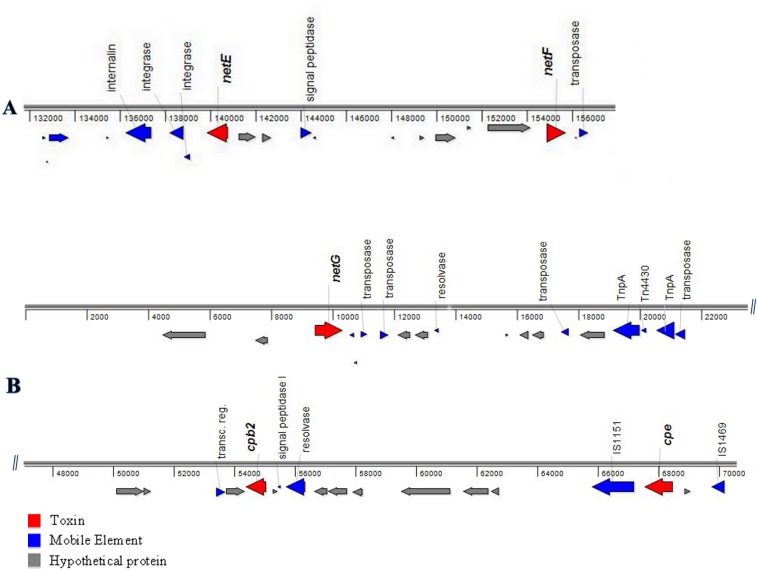
Genetic organization of JFP718 scaffolds. The genetic organization of (A) partial view of scaffold00006 (GenBank KP739975), (B) partial view of scaffold00012 (GenBank KP739976) is shown, each arrow representing a predicted gene and its orientation. Predicted functional annotations and respective positions are shown above each gene, respectively. Genes are color-coded by their putative role based upon sequence analyses.

*C*. *perfringens* strain JFP718 was isolated from a case of fatal canine hemorrhagic gastroenteritis diagnosed in a 2-year-old female Pomeranian dog that was found dead the day after attending a dog show [13]. The strain was sequenced because of both the classic appearance of the disease in this dog and the highly cytotoxic effect of its supernatant. The automated annotation of the draft genome sequences of *C*. *perfringens* canine strain JFP718 were essential to the discovery of the three putative toxin genes, initially denominated as Panton-Valentine leukocidins (PVLs). Two PVL open reading frames (ORF) were located in scaffold00006 (nucleotides 139809–140777 [–] and 154804–155721[+]) (GenBank KP739975), while the third was in scaffold00012 (nucleotides 9399–10319 [+]) (GenBank KP739976), both surrounded by transposon-related sequences. Interestingly, both scaffolds (00006 and 00012) contained mainly plasmid genes, suggesting that these sequences were associated with a transposable element and/or plasmid. The newly identified toxin genes were named *netE*, *netF* and *netG*. The predicted proteins (NetE, NetF and NetG) encoded by these three ORFs showed 79%, 48% and 52% identity, respectively, with the pore-forming toxin NetB from *C*. *perfringens* (EU143239) (Table 1 and S1 Fig). Scaffold 00006 harbors *netE* and *netF* toxin genes among 140 ORFs. Immediately upstream of the *netE* and *netF* genes, which are 14,027 nt apart, are two mobile element proteins classified as an integrase (*C*. *botulinum* BKT015925) and a transposase (*C*. *perfringens*), whereas downstream of the *netF* gene there is another integrase (*C*. *botulinum* BKT015925) (Fig 1A). Scaffold 00012 harbors the *netG* gene as well as the *cpb2* (CPB2 toxin) (54351–55052) and *cpe* (67515–68474) genes (Fig 1B).

**Table 1:** Characterization of Net toxins.

Protein^1^	Length (aa)	Molecular size (mature protein) in kDa	Predicted Product	E-Value	% of Identity^2^	Localization^3^	Conserved Domain	Accession	PSSM-ID	SignalP^4^
NetE	322	36.1 (32.9)	Putative beta-pore-forming toxin	3.14E-71	254/322 (79%)	Extracellular	Leukocidin/Hemolysin toxin family	cl08468	244969	30–31
NetF	305	34.3 (31.7)	Putative beta-pore-forming toxin	4.41E-57	143/299 (48%)	Extracellular	Leukocidin/Hemolysin toxin family	cl08468	244969	24–25
NetG	306	34.3 (31.7)	Putative beta-pore-forming toxin	2.89E-70	143/276 (52%)	Extracellular	Leukocidin/Hemolysin toxin family	cl08468	244969	24–25

^1^Based on strain JFP718 genome

^2^Percent amino acid identity with NetB (query length/total length of the subject protein)

^3^Subcellular location as predicted by pSortb

^4^Signal peptide was predicted by SignalP cleavage position

**Fig 2:**
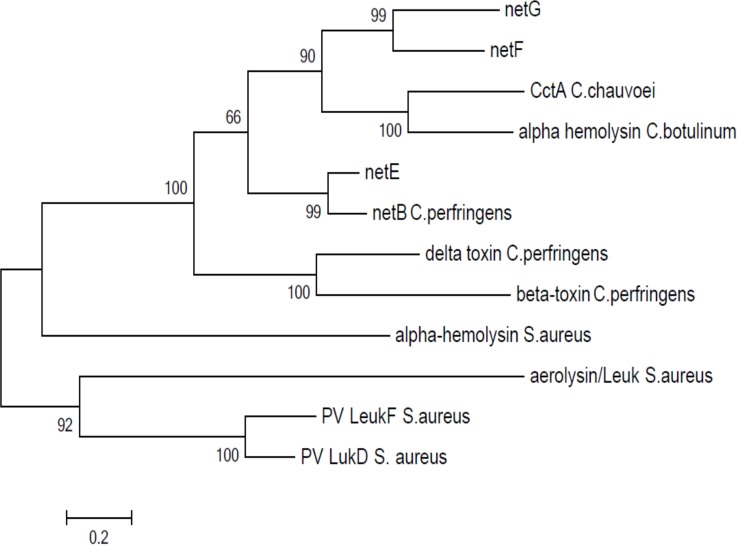
Phylogenetic analysis of representative members of the Leukocidin/Hemolysin superfamily. The phylogenetic tree was built by the Neighbor-joining algorithm using (1000 interactions) MEGA5 software (35). The tree is drawn to scale, with branch lengths in the same units as those of the evolutionary distances used to infer the phylogenetic tree. Toxins that were used included: alpha-hemolysin of *C*. *botulinum* (YP_004394739.1), hemolysin II of *B*. *cereus* (YP_002447023.1), alpha-hemolysin of *S*. *aureus* (WP_001788633), putative CctA of *C*. *chauvoei* (WP_021874975) and beta-toxin of *C*. *perfringens* (CAA58246.1).

Sequence analyses of these ORFs were performed using BLAST, BLASTP, the Conserved Domains Database (CDD) [31], SignalP [32], pSortB [33], and InterProScan [34] (Table 1). Analysis against the CDD conserved domain database showed that these newly described *net* genes encode proteins belonging to the Leukocidin/Hemolysin superfamily. InterProScan analysis and classification also confirmed the presence of the Leukocidin/Hemolysin domain (IPR001340) and classified them as members of the alpha-hemolysin branch of the beta-pore-forming toxin family. The new genes are predicted by pSortB and SignalP to encode extracellular proteins. *netE* encodes a putative 322 amino acid (aa) protein with a signal peptide region of 30 residues, *netF* encodes a 305 aa protein, and *netG* encodes a 306 aa protein, both with a signal peptide region of 24 residues. The predicted respective molecular size of the mature NetE toxin is 32.9 kDa, and of NetF and NetG are 31.7 kDa. The close relationship of the novel Net toxins to other members of the Leukocidin/Hemolysin pore-forming toxin family is shown through the Clustal W alignment (S1 Fig) and phylogenetic tree (Fig 2) [35]. For NetE, the closest ortholog (79% identity) is NetB from *C*. *perfringens*, whereas NetF and NetG were placed in their own branch (Fig 2).

The VirR/VirS two-component signal transduction system regulates several virulence genes in *C*. *perfringens*, including *cpa* (alpha-toxin or phospholipase C), *cpb* (beta-toxin), *pfoA* (perfringolysin O or theta-toxin), *colA* (kappa-toxin or collagenase), and *netB* [36,37]. Analysis of the upstream region of *net* genes revealed the presence of potential VirR boxes [38] in the promoter region of *netE* and *netG*, but not of *netF* S1 Table.

### Cytotoxicity of *Clostridium perfringens* Type A Isolates

To determine whether *C*. *perfringens* type A strains isolated from fatal cases of canine hemorrhagic enteritis and foal necrotizing enteritis produced a distinct secreted toxin, sterile culture supernatants of strains JFP718 (canine origin) and JFP728 (equine origin) were tested for cytotoxicity against 10 cell lines derived from 9 animal species (Table 2). The culture supernatants of the two strains showed potent cytotoxic effects for an equine ovarian cell line (EO) at a dilution of 1:128, with far less or no toxicity against other cell lines tested (Table 2). By contrast, supernatants derived from NCTC3110 (*cpa*, *cpb* positive), JFP564 (*cpa*, *cpb2* and *cpe* positive), SM101 (*cpa*, *cpe* positive) and CW504 (*cpa* positive) displayed mild or no cytotoxic effects on EO cells, suggesting that toxicity of JFP718 and JFP728 strains might be caused by a secreted component(s) distinct from other known toxins (Table 3).

**Table 2:** Cytotoxicity of different cell lines with bacterial culture supernatant of *netF-positive* (JFP726, JFP728) and *cpb*-positive (NCTC3110) *C*. *perfringens* strains.

		Cytotoxicity titer^2^		
Cell lines^1^	Origin of cell lines	NCTC3110	JFP728 (Equine)	JFP726 (Canine)
EO	Equine	16	128	128
MDCK	Canine	N^3^	4	4
A72	Canine	4	8	8
MDBK	Bovine	N	2	2
PK15	Pig	N	4	4
208F	Rat	2	2	2
NIH 3T3	Mouse	N	N	N
CaCo2	Human	N	2	2
LMH	Chicken	2	N	N
Vero	Monkey	N	N	N

^1^EO: Equine ovarian cell line, MDCK: Madin Darby canine kidney cell line, A72: Canine fibroblasts cell line, MDBK: Madin Darby bovine kidney cell line, PK15: Porcine kidney cell line, 208F: Rat Fischer fibroblast cell line, N1H 3T3: Mouse embryo fibroblast cell line, CaCo2: Human colon epithelial cell line, LMH: Primary chicken hepatocellular carcinoma epithelial cell line, Vero: African green monkey kidney cell line.

^2^ Cytotoxicity was evident after 8 h of exposure of 2-fold dilution series of the filter-sterilized culture supernatants up to 1:1024 to different cells. The titer is the final dilution showing > 2+ cell toxicity.

^3^ No cytotoxicity.

**Table 3:** Cytotoxicity of equine ovarian cell line (EO) with different bacterial culture supernatants.

Strains (toxin genes)^1^	Cytotoxin titer^2^
NCTC3110 (*cpb*+)	16
JFP564 (*cpe*+/*cpb2*+)	8
SM101 (*cpe*+)	4
JFP728 (*netE/F/G*+)	128
JFP718 (*netE/F/G*+)	128
CW504 (*cpa*+)	N^3^
TPG broth control	N

^1^NCTC3110: type B, JFP564: type A, SM101: Derivative of NCTC 8798, JFP728: equine necrotizing enteritis, JFP718: canine hemorrhagic enteritis, CW504: lab strain. All strains possessed the *cpa* gene. Toxin titers were assessed over 10 times.

^2^Dilution of filter-sterilized bacterial supernatants from TPG broth cultures with an OD_600_ of 0.6–0.8 that showed end-point > 2+ cytotoxicity

^3^No cytotoxicity

### Characterization of Large Plasmids by PFGE Analysis and Southern Blots (SB)

**Fig 3:**
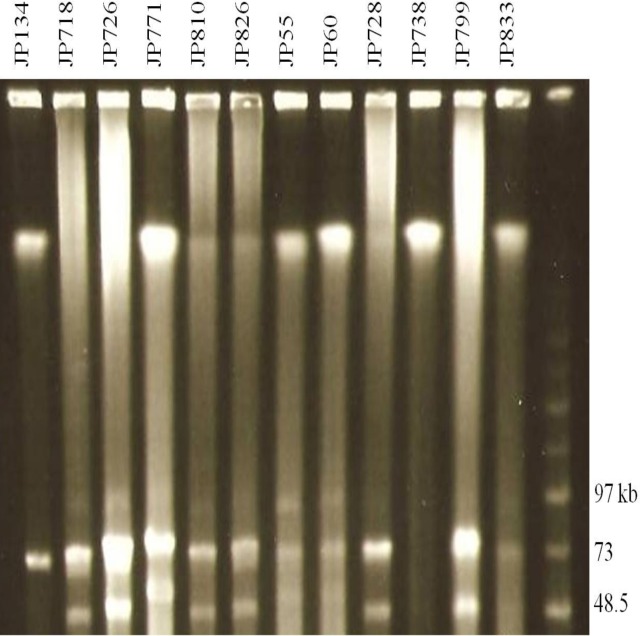
PFGE analyses of plasmids from canine and equine *C*. *perfringens* strains. Agarose plugs containing DNA from each specified isolate were digested with *Not*I and subjected to PFGE and staining with ethidium bromide. Line numbers indicate isolate numbers; M: Mid-Range II PFG molecular DNA ladder (Kb).

**Fig 4:**
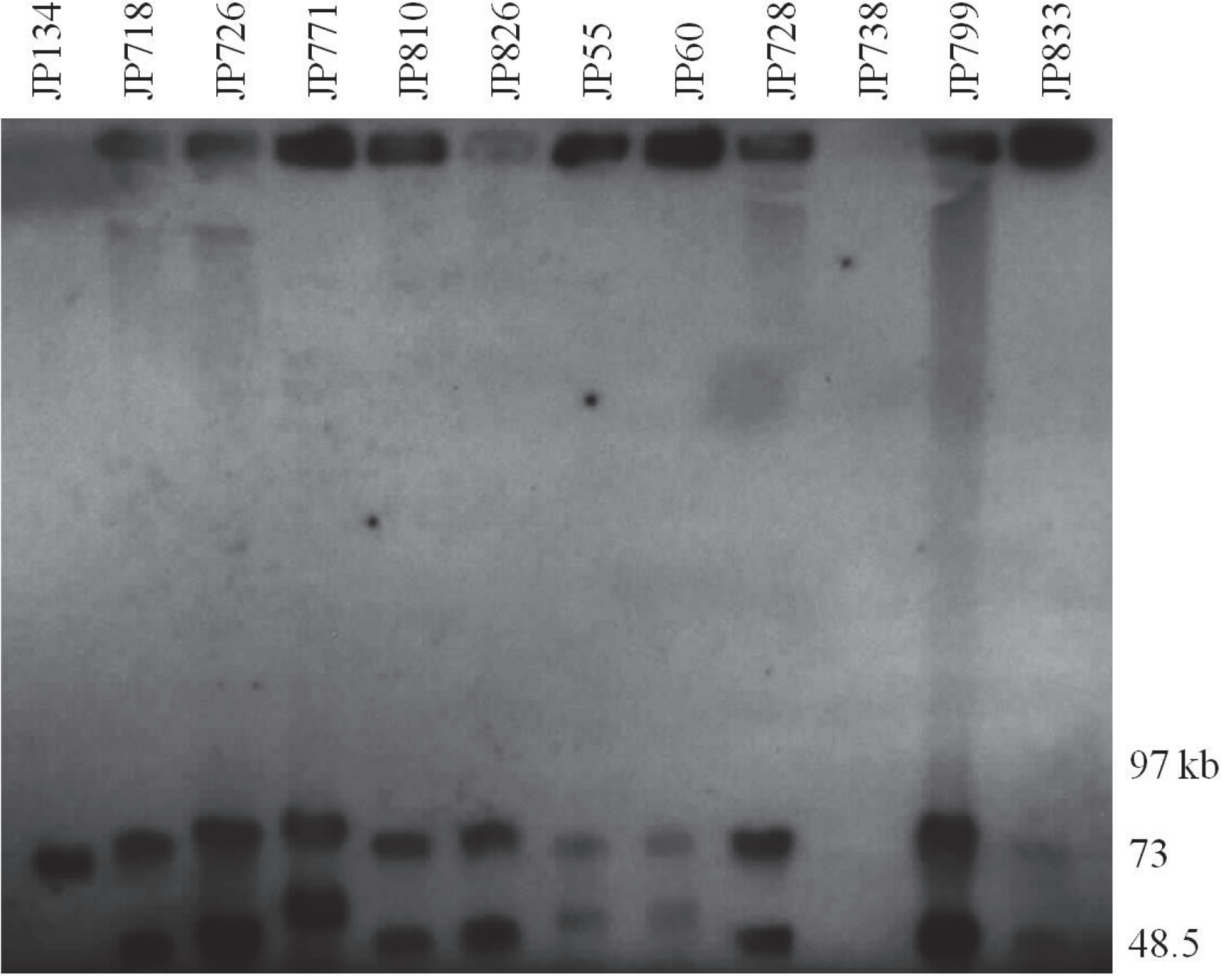
PFGE-Southern blot of plasmids from canine and equine *C*. *perfringens* strains. Southern blotting of PFGE was performed with DIG-labelled probes for *netE* and *cpe* genes. Results from both *netE* and *cpe* probes are shown overlayed. In all lanes with two bands, the upper band represents *netE* and the lower band *cpe*. M: Mid-Range IIPFG molecular DNA ladder (Kb).

To determine the presence of large plasmids in equine and canine type A *C*. *perfringens* isolates, genomic DNA from six canine and six equine isolates (Table 4) were subjected to PFGE. The PFGE profiles of the canine and equine *C*. *perfringens* strains digested with *Not*I revealed the presence of two to three large plasmids ranging in size from 45–97 kb in all strains (Fig 3 and Table 5). PFGE analysis (Fig 3) shows the diversity of plasmids among these type A isolates and their marked size variations, which were confirmed by PFGE/SB studies (Fig 4). Specifically, the partially sequenced canine type A *C*. *perfringens* JFP718 strain carried three large plasmids (Fig 3) in which the *netE* and *cpe* genes were on a distinct plasmid (Fig 4). The SBs showed the presence of *tcpF* in all the large plasmids (data not shown). In addition, *cpb2* was identified by SB in the *netG* plasmid as described in the scaffold 00012 (data not shown) confirming the hypothesis that these two scaffolds represent two distinct plasmids. Interestingly, the non-cytotoxic strains JFP134 (canine) and JFP738 (equine) which showed one and no large plasmids, respectively, were also SB negative for *netE* (Fig 4).

**Table 4:** Bacterial strains used in this study.

Strain	Name	Relevant characteristics	Source
***C*. *perfringens***	NCTC3110	Type B	NCTC, UK
	NCTC7368	Type B	NCTC, UK
	NCTC3181	Type C	NCTC, UK
	ATCC3628	Type C	ATCC, USA
	SM101	Derivative of NCTC 8798	J.Gong, AAFC
	CW504	Rif^R2^ Nal^R^; conjugation recipient	J.I.Rood, Monash University
	JIR325	Strain 13 Rif ^R^Nal^R^; electroporation recipient	J.I.Rood, Monash University
	Strain 33	*C*. *perfringens* strain 33 was used to normalize the gel and allow for gel-to-gel comparisons	This study
	CP1	*netB*+ *C*. *perfringens*/Chicken necrotic enteritis	[39]
	CP4	*netB*+ *C*. *perfringens*/Chicken necrotic enteritis	[39]
	JFP55	Equine strain/Undifferentiated diarrheal disease	This study
	JFP60	Equine strain/Undifferentiated diarrheal disease	This study
	JFP134	Canine strain/Undifferentiated diarrheal disease	This study
	JFP564	Human strain/ Type A	This study
	JFP718	Canine strain/Hemorrhagic gastroenteritis	This study
	JFP726	Canine strain/Hemorrhagic gastroenteritis	This study
	JFP728	Equine strain/Necrotizing enteritis	This study
	JFP738	Canine strain/Undifferentiated diarrheal disease	This study
	JFP771	Canine strain/ Hemorrhagic gastroenteritis	This study
	JFP799	Equine strain/Necrotizing enteritis	This study
	JFP810	Canine strain/Hemorrhagic gastroenteritis	This study
	JFP826	Canine strain/Hemorrhagic gastroenteritis	This study
	JFP833	Equine strain/Necrotizing enteritis	This study
	JFP838	Equine strain/Hemorrhagic enterocolitis	This study
	JFP838E-05	JFP838::*netE*::ErmRAM—ClosTron insertion in *netE* gene	This study
	JFP838F-05	JFP838::*netF*::ErmRAM—ClosTron insertion in *netF* gene	This study
	JFP838G-07	JFP838::*netG*::ErmRAM-ClosTron insertion in *netG* gene	This study
	T504-05::*netE*	CW504 derived transconjugant Rif^R^Nal^R^Erm^R^with plasmid pNetE/NetF from JFP838E-05	This study
	JFPnetF	JIR325 derived transconjugant Rif^R^Nal^R^Cm^R^ with plasmid pNetF07	This study
	VN-22C	JFP838F-05 Erm^R^Cm^R^ complemented with pNetF07	This study
***E*. *Coli***	DH5α	*F* ^*-*^ *Φ80 lacZΔM15Δ (lacZYA-argF) U169 endA1 recA1 hsdr17 (r*_*K*_^*-*^ *m*_*K-*_ *) deoR thi-1 supE44 gyrA96 relA1*	Stratagene, La Jolla, CA
	BL21-Star (DE3) pLysS	*E*. *coli B F* ^*—*^ *dcm ompT hsdS (r*_*B*_^*—*^ *m*_*B*_^*–*^ *) gal λ(DE3)* [pLysS Cam^R^]	Invitrogen
	CA434	*E*. *coli* HB101 carrying the Incβ conjugative plasmid R702	[40]; G. Vedantam, University of Arizona
	CA434-netE	*E*. *coli* CA434 carrying plasmid pMTL::*netE*	This study
	CA434-netF	*E*. *coli* CA434 carrying plasmid pMTL::*netF*	This study
	CA434-netG	*E*. *coli* CA434 carrying plasmid pMTL::*netG*	This study

**Table 5:** Features of type A canine and equine *Clostridium perfringens* strains.

		PCR^1^			Southern blot^2^	
Strains	Plasmid number	*netF/netF*	*netG*	*cpe*	*netE*	*cpe*
JFP134	1	-	-	+	-	70
JFP718	3	+	+	+	75	45
JFP726	3	+	+	+	75	48
JFP771	3	+	-	+	75	50
JFP810	2	+	+	+	75	48
JFP826	2	+	+	+	75	48
JFP55	3	+	-	+	75	50
JFP60	3	+	-	+	75	50
JFP728	2	+	+	+	75	48
JFP738	-	-	-	-	-	-
JFP799	2	+	+	+	75	48
JFP833	2	+	-	+	75	48

^1^Genes detected by PCR amplification (-) negative and (+) positive

^2^Approximate size of plasmids in kb

### PFGE Analysis of *netF*-Positive and Negative in Canine and Equine Type A *C*. *perfringens*

**Fig 5:**
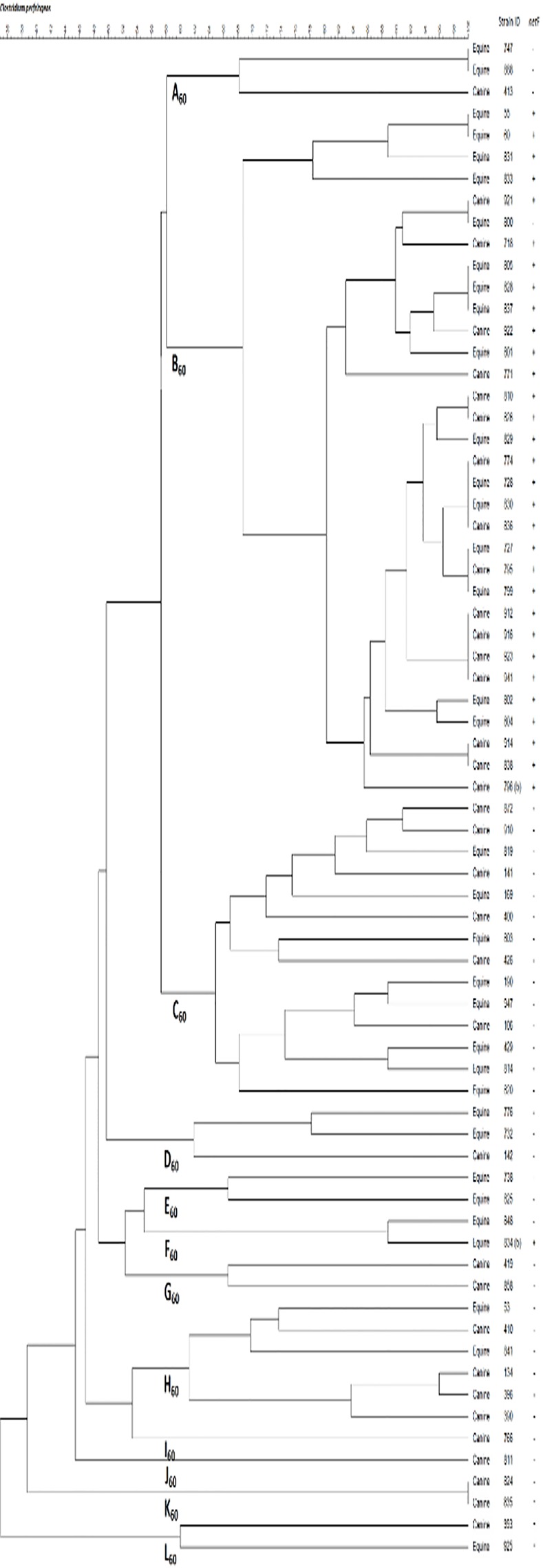
Dendogram of *Clostridium perfringens* isolates. Dendogram of *C*.*perfringens* isolates typed by pulsed-field gel electrophoresis and analysed using BioNumerics software. The BioNumerics software used was version 7.1 from Applied Maths, Austin, TX.

There is increasing evidence that *C*. *perfringens* associated with different enteric diseases may belong to clonal populations. We used PFGE to examine the relatedness of canine and equine *netF+* and *netF-* isolates from our strain collection. The PFGE analysis and the dendogram are shown in Fig 5. Out of the 70 isolates (35 equine and 35 canine) typed by PFGE, 56 (80%) individual pulse types were identified based on an identical banding pattern (genetic similarity of 100%). Eighteen of these 56 were positive for *netF*, 37 were negative for *netF*, and one type contained both *netF*+ and *netF*- isolates that were equivalent. There was no pattern distinguishable between equine and canine types (Fig 5).

Using the criterion of 60% genetic similarity, 11 clades (A to L) were distinguished (Fig 5). All but one of the *netF*+ strains belonged to one clade when using this relatedness criterion (Fig 5). The *netF*- isolates comprised a wider diversity of clades (Fig 5). Only one *netF*+ isolate, JFP834, was included in one of these *netF*- clades. Using a less stringent genetic similarity cut-off of 70%, all except one of the *netF+* strains belonged to two clades whereas the *netF-* strains belonged to 21 clades.

### Mutation of *netE*, *netF* and *netG* and Conjugation

In order to confirm which one of the Net proteins was responsible for cytotoxicity on EO cells, several assays were performed.

Firstly, the *netE*, *netF* and *netG* genes were insertionally inactivated in strain JFP838 by the ClosTron method, resulting in the mutant strains JFP838E-05, JFP838F-05 and JFP838G-07, respectively. The insertion of ErmB-carrying introns into each of the three target genes was confirmed by Southern blot and PCRs. Southern blot assay using specific intron probe (S2 Table and S2 Fig) showed the insertion of the intron in the target sites as well in other sites in the case of the *netF* and *netG* mutants (S2 Fig). The insertion of ErmB-carrying introns into each of the three target genes was confirmed by two PCRs, one using specific primers flanking the insertion site (*netE*, *netF* and *netG* primers) and another using a combination of specific primers with intron primer (EBS-universal) (S2 Table and S3A and S3B Fig). The mutant strains JFP838E-05 (::*netE*) and JFP838G-07(::*netG*) retained wild-type cytoxicity on EO cells, whereas the JFP838F-05 (::*netF*) mutant was not cytotoxic.

**Fig 6:**
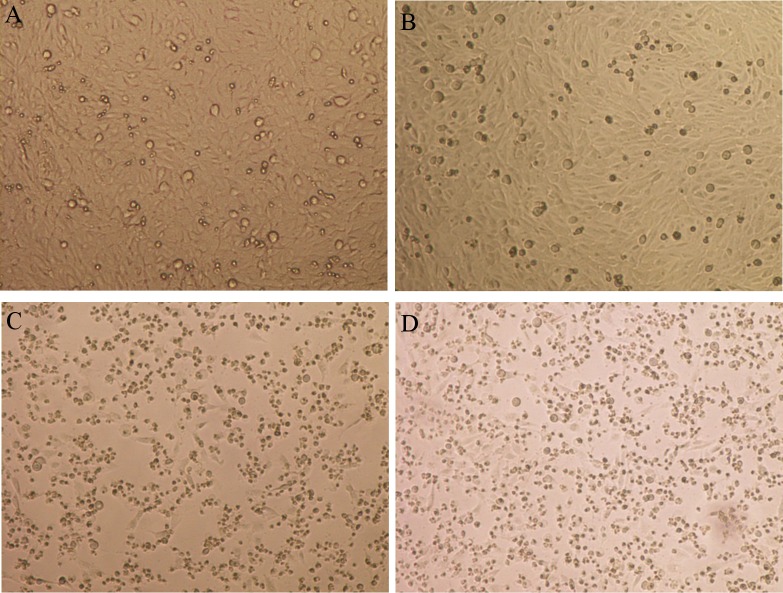
Infection and cytotoxic effects on equine ovarian (EO) cells by supernatant from JFP838 and its isogenic derivatives. Confluent EO cell cultures were infected for 8 h at 37°C/5%CO2. Filter-sterile broth culture supernatants were used for these infections: (A) Typical morphology of EO cells, (B) JFP838-F05 (*netF* null mutant), (C) Wild-type JFP838, (D) Complementing strain VN-22C. Cytotoxic effects to EO cells in these conditions were cell rounding, detachment and death of cell in the cell plate, seen in Fig 6C and 6D. Magnification×100.

Secondly, the non-cytotoxic mutant strain JFP838F-05 was complemented *in trans* with pNetF-07, which contains the entire *netF* gene. The *netF* gene in pNetF-07 restored the cytotoxicity of the JFP838::*netF* mutant (JFP838F-05) similar to that of the wild-type JFP838 (Fig 6). Western blot attested to the expression of NetF in the complemented but not the mutant strain (S4 Fig).

Thirdly, conjugation assays were performed using mutant strains (JFP838E-05 and JFP838F-05) as host and the laboratory strain CW504 as recipient, where plasmids (pCP718netF and pCP718cpe) were transferred to the recipient strain. The erythromycin-resistant transconjugants were confirmed by specific PCR amplifications of *netE*, *netF* and *netG* genes; the *ermB* gene was amplified from the transconjugants but not from the wild-type or recipient strain (Table 4). The transconjugant T504-05::*netE*, which harbored only the pCP718netF::*netE* plasmid, and not the pCP718cpe plasmid containing *netG* as confirmed by PCR (data not shown), showed cytotoxicity similar to that of the wild-type JFP838 strain. This evidence also suggested that the NetF toxin was producing the cytotoxic effect.

Finally, to confirm that the cytotoxicity was related entirely to NetF and not to any other protein expressed by the pCP718netF plasmid containing *netE* and *netF*, the pNetF-07 plasmid, which harbors the entire *netF* gene, was electroporated into laboratory strain *C*. *perfringens* JIR325. This recombinant strain, named JFPnetF, which expressed the NetF toxin but not the other Net toxins, was found to have cytotoxicity for EO cells similar to that of wild-type strain JFP838, whereas the laboratory strain JIR325 used as control was not cytotoxic.

Taken together, the results described above are clear evidence that the cytotoxic effect observed is produced by *netF* and that, of the three *net* genes found in these cytotoxic strains, *netF* alone is responsible for the cytotoxic activity.

### Prevalence of *net* Genes and their Association with Foal Necrotizing Enteritis and Canine Hemorrhagic Enteritis, and with Cytotoxicity

The *netF* gene was identified in 11 of 15 isolates (74%) from different foals with fatal necrotizing enteritis whereas it was not found in 11 foals with undifferentiated diarrheal disease (*p*<0.00019; MUE = 31.45, CI = 5.35-∞). The *netF* gene was only identified in 4 of 58 (6.8%) isolates from adult horses with undifferentiated diarrheal disease (*p*<0.0000004 compared to foals with fatal necrotizing enteritis; CMLE≃33.63, CI = 6.58–170.95). In canine fatal hemorrhagic gastroenteritis, the *netF* gene was found in 8 of 11 isolates (73%) compared to 8 of 81 (10%) undifferentiated canine diarrheal isolates (*p*<0.000000081; CMLE≃40.44, CI = 8.01–204.72).

PCR analysis of equine and canine *netF*-positive isolates (n = 31) showed that the *netF* gene was always (100%) found in association with both the *netE* gene and the *cpe* enterotoxin gene, but in fewer (55%) isolates with the *netG* gene. *netG* was found in 5 of 11 (46%) isolates from canine hemorrhagic gastroenteritis compared to 7 of 81 (9%) isolates from dogs with undifferentiated diarrheal disease (*p*<0.0049; CMLE≃8.46, CI = 2.05–36.36). *netG* was only identified in 7 of 15 (47%) of foal necrotizing enteritis isolates compared to none of 11 foals with undifferentiated diarrheal disease (*p*<0.01; MUE = 11.15, CI = 1.9-∞) and none of 58 adult horses with undifferentiated diarrheal disease (*p*<0.000004, MUE = 59.95, CI = 10.75-∞). None of 24 caprine, 28 ovine and 47 bovine different source isolates tested was *netE*, *netF* or *netG* positive. Subsequent PCR amplifications of the *netE*, *netF* and *netG* genes from three canine hemorrhagic enteritis isolates and three other foal necrotizing enteritis isolates and DNA sequencing showed these genes to be fully conserved at the nucleotide level.

The supernatant of 29 of 31 canine and equine *netF*-positive isolates was as toxic as that of JFP728 for the EO cell line. Of 176 different *C*. *perfringens* type A isolates tested, 29 of 31 *netF*-positive strains were both cytotoxic and immunoblot positive using NetF antiserum compared to none of 145 *netF*-negative enteric strains of avian, bovine, canine, caprine, equine or human origin tested from our strain collection (details not given); immunoblots of the two non-toxic *netF*-positive strains revealed that NetF was not produced in these two strains (data not shown).

### Recombinant Net Toxins

Recombinant Net toxins (rNetE, rNetF and rNetG) were engineered without the signal peptide (SP) sequence and, because of solubility problems, were purified under denaturing conditions using 8 M urea. To obtain soluble recombinant Net toxins, rNet proteins were constructed with the *E*. *coli* fusion protein NusA (45 kDa). SDS-PAGE and immunoblot results showed that the fusion proteins were expressed at a high level in a soluble form (data not shown).

#### Cytotoxin Neutralization by Equine Polyclonal Antibodies

Immunoblotting of SDS-PAGE separated rNetE-NusA, rNetF-NusA or rNetG-NusA showed cross-reactivity of sera prepared against each of the recombinant proteins (data not shown). Cross-reactivity similar to that noted in immunoblotting was also apparent in ELISA when these sera were tested against individual rNet-NusA proteins (Table 6), with the titer (and immunoblot cross-reactivity) being highest against the homologous protein. Following immunization, only horses immunized with rNetF-NusA showed a high degree of cytotoxin neutralizing activity when tested against supernatant from wild-type (*netE/F/G*) positive strains (Table 6).

**Table 6:** ELISA cross-reactivity and cytotoxin neutralizing titers to recombinant toxins rNetE, rNetF and rNetG in horses immunized with different rNet-NusA proteins.

			ELISA			
Antigens	Horses^1^	Neutralizing anti-serum titers^2^	rNetE	rNetF	rNetG	rNetB
rNetE	1	643	102400	6400	12800	51200
	2	128	25600	1600	1600	12800
rNetF	3	25600	12800	204800	12800	6400
	4	6400	6400	102400	12800	12800
	5	3200	12800	204800	12800	6400
rNetG	6	64	6400	12800	51200	12800
	7	128	51200	51200	102400	51200

^1^Immunization: Horses 1, 2, rNetE-NusA; horses 3–5, rNetF-NusA; horses 6, 7, rNetG-NusA

^2^The neutralization of cytotoxicity by polyclonal antibody was performed with the EO cell line

^3^The neutralizing antibody titer was that showing an inhibition of 2+ or greater

To determine the specificity of antibodies produced in horses to the individual rNet-NusA proteins, immunoblot of culture supernatants of two type B (*cpb*-positive), two type C (*cpb*-positive) and two type A *netB*-positive strains was performed using horse sera prepared against rNet-NusA proteins. Antisera prepared against each of the recombinant proteins interacted weakly with NetB but not with the CPB toxin in type B or C strains (data not shown).

## Discussion

This paper advances understanding of type A *C*. *perfringens* as an enteric pathogen of animals by identifying a novel pore-forming toxin within the Leukocidin/Hemolysin superfamily family, NetF, and the association of a clonal population expressing this toxin with two distinct severe enteric diseases, canine hemorrhagic gastroenteritis and foal necrotizing enteritis.

A partial genome sequence identified three distinct genes (named *netE*, *netF* and *netG*) within the Leukocidin/Hemolysin pore-forming superfamily to which the α-toxin of *Staphylococcus aureus* and the CPB and NetB toxins of *C*. *perfringens* belong. Pore-forming toxins (PFTs) to which these new Net proteins belong, are potent cytolytic agents secreted by pathogenic bacteria that protect microbes against the cell-mediated immune system, disrupt epithelial barriers, and liberate nutrients necessary for growth and colonization. *C*. *perfringens* type A strains isolated from fatal cases of canine hemorrhagic enteritis and foal necrotizing enteritis produced secreted cytotoxin(s) toxic for an equine ovarian cell line (EO). The challenge was to determine whether one or all of the Net toxins was responsible, since immunoblotting showed that all three proteins were expressed (S4 Fig).

A series of mutations and conjugation assays proved that NetF was responsible for the marked cytotoxicity of EO cells. Mutations in the *netE* and *netG* genes did not cause any reduction in the cytotoxicity activity; only mutation of *netF* was able to eliminate the cytotoxic effect. Despite the fact that another two sites had intron insertions in the *netF* mutant, the complementation of the *netF* mutant by the *netF* gene completely restored the cytotoxic activity for EO cells (Fig 6). Two other approaches were also used to confirm NetF as the toxin responsible for cytotoxicity, firstly by transferring the pCP718netF plasmid containing *netE* and *netF* from JFP838E-05 (mutated *netE*) to *C*. *perfringens* CW504 by conjugation and secondly by electroporating the shuttle vector with the entire *netF* gene (pNetF-07) to *C*. *perfringens* JIR325. In both cases, bacterial supernatants produced cytotoxicity on EO cells equivalent to wild-type (JFP838). In addition, only antiserum against recombinant NetF protein neutralized the cytotoxicity of supernatants at high titer (Table 6).

The particular susceptibility of the EO cells compared to other cell lines (Table 2) may relate to specific receptors or factors other than those relating simply to the animal host of origin of the cell line. Others have found marked differences between chicken cell lines in susceptibility to NetB, a related pore-forming toxin important in the pathogenesis of necrotic enteritis of chickens [2]. Further work is required to identify the receptor for NetF.

Although *netE* and *netG* are expressed (S4 Fig), we found no evidence that they had cytotoxic activity. It is possible that they might contribute to the ability to cause disease in hosts other than dogs and horses. An alternate possibility is that NetE and NetG might have cytotonic effects, such as proinflammatory effects, as has been shown with pore-forming Leucocidins of *Staphylococccus aureus* [41]. There may also be synergism with other toxin molecules [41]. The consistent presence of *cpe* on a separate large plasmid in the *netF*-positive strains is an intriguing finding. A recent study of a type C human enteritis necroticans strain, and of purified CPB and CPE, showed the marked synergistic interaction of small amounts of these two toxins in causing fluid accumulation and histologic damage the intestine of rabbits [42].

The consistent presence of *cpe* in *netF*-positive strains is indirect evidence that CPE may contribute to the disease process in dogs and foals. CPE was not produced under the growth conditions used in this study (S4 Fig) but speculatively may have an important synergistic interaction with NetF in promoting disease *in vivo*. It is also possible that NetE and NetG might synergize with CPE in producing intestinal toxicity *in vivo*. *netG* was however present in just over half of the strains carrying *netE*, *netF*, and *cpe*, suggesting that its role in canine hemorrhagic enteritis and foal necrotizing enteritis is relatively unimportant.

Pore-forming toxins are released as water-soluble monomeric or dimeric species, bind specifically to a variety of receptors in membranes, and assemble transmembrane channels leading to cell damage and commonly to lysis [43]. De and Olsen [43] have shown that conservation of many of the key amino acids seems to be essential for their pore-forming function S1 Fig. Savva *et al*. [44] have identified conserved and non-conserved amino acid positions that affect binding and toxicity of NetB. Since the tyrosine at position 202 (NetB:Y202) is only conserved in the clostridial pore-forming toxins, Savva *et al*. [44] suggested that its proximity to Arg-200 may contribute to an interaction with the glycerol backbone, which reduces the hemolytic activity of NetB Y202A. In NetF, the tyrosine is not conserved, but rather histidine is present at the equivalent position S1 Fig. Yan *et al*. [45] proposed that the serine at position 254 (S254) of NetB is essential for the formation of an oligomer on the surface of the target cell. Notably, there is a substitution of S245T in NetF which could also impact the oligomerization of this toxin. The clostridial and staphylococcal members of the β-PFTs family may bind to host membranes using different mechanisms involving interactions with different lipids or proteins. The structure of the β-PFTs shows a ring-shaped complex which resembles a mushroom; on the rim domain, Trp-257 and Trp-262 have been shown to be involved in cell binding of NetB [44, 45]. Substitution to alanine at either position (W257A and W262A) reduced NetB cytotoxicity and hemolysis on LMH cells and on RBCs, respectively. Da Costa *et al*. [46] showed the importance of lysine at position 41 (K41) for oligomerisation and pore-formation of NetB. These residues are conserved in β-barrel PFTs of *S*. *aureus* and *C*. *perfringens*, as well as in NetE, NetF and NetG S1 Fig. The identification of three new members of the β-barrel PFT family may provide additional proteins with which to explore the receptor-based selectivity and specificity of members of this family [45,47].

Clonal expansion and niche specialization is well recognized in particular types of *C*. *perfringens* [48], such as *cpe*-bearing type A food poisoning isolates [9], porcine *cna* and *cpb2*-containing isolates [49], and *cpb2* and *netB*-containing poultry isolates [7,50]. The clonality of the canine and equine *netF*-positive strains identified here, and the lack of any immediately obvious common infectious source relationship between dogs and neonatal foals, might suggest that they are infected by strains adapted to a common environmental source rather than to dogs or horses. The close relatedness, or in some cases the identity, of canine and equine isolates identified by PFGE was mirrored in the complete nucleotide conservation of the *net* genes, though size differences were noted in the plasmids encoding these genes (Fig 3). Additional studies will determine which are the fully conserved regions of these plasmids, and whether these plasmids have common pathogenicity loci [4,51].

The virulence of *C*. *perfringens* is dependent on its remarkable ability to produce a variety of toxins, of which some of the most toxic are found on the large family of conjugative *tcp* plasmids [4,52,53]. Although strains may carry up to three different large toxin plasmids [5,51], we found only two in the majority of pCP718netF-positive isolates, one carrying *netE* and *netF*, and the other consistently carrying the *cpe* enterotoxin gene and often the *netG* gene. *C*. *perfringens* virulence plasmids share a conserved backbone sequence which contains among other genes the *tcp* conjugation locus [4]. The *tcp* locus is present on all known conjugative plasmids from *C*. *perfringens* and consists of 11 genes (*tcpA* to *tcpJ*), of which two (*tcpF*, *tcpH*) are essential for conjugative transfer [54]. pCP718netF and pCP718cpe plasmids carried *tcpF* genes as shown by SB, but further work is required to show whether these are both conjugative. This study has added to understanding of the role of plasmids in the adaptability and flexibility of virulence in *C*. *perfringens* [53]. The likely basis of the presence of independently conjugative plasmids with large common genetic regions has been suggested [52] and identified [4,8]. Although toxin gene-bearing large plasmids are critical in the virulence of *C*. *perfringens*, the chromosomal background in which these plasmids exist may enhance both the host specificity and the virulence of the strain [7,8,9]. The *netF*-positive strains were almost entirely clonal (Fig 5), suggesting that the chromosomal background is important for maintenance of these plasmids and/or for virulence. A feature of the *netF*-positive strains identified here was that they always contained one plasmid with *netE* and *netF* and another with *cpe* and usually, but not always, with *netG*. The relatedness of strains carrying a plasmid-encoded *cpe* has been noted previously [55] but not at the degree of conservation identified here.

Many toxin genes in *C*. *perfringens* are positively regulated at exponential phase by the two-component VirR/VirS that is a major regulator of virulence in *C*. *perfringens* [36], including production of NetB system toxin by chicken necrotic enteritis strains [37] and CPB in type C strains [36]. We found that *netE* and *netG* genes have a putative VirR box upstream of their start site, and it is therefore possible that these toxin genes are regulated by the VirR/VirS system as are many other *C*. *perfringens* toxins. Obana and Nakamura [56] have shown that CPE1447 and CPE1446 controls target genes as novel transcriptional regulators. This might also be the case in the regulation of NetF toxin, or alternatively it may be regulated by VirR-regulated RNA [57].

Canine hemorrhagic gastroenteritis has long been a poorly understood disease of dogs, known to be often associated with *C*. *perfringens* and characterized by its dramatic and sometimes fatal nature [14,15]. The pathology of fatal cases of *C*. *perfringens*-associated canine hemorrhagic gastroenteritis is characterized by coagulative necrosis in the small intestine, a disease process typically associated with pore-forming toxins in *C*. *perfringens*. This study associates *netF*-producing strains with this disease, and will contribute to improved diagnosis, treatment and control. In the light of the current advance in understanding the basis of canine hemorrhagic gastroenteritis, the well-recognized association of this disease with small breed dogs [14,15,58] may be explained by the increased incidence of pancreatitis in small rather than in large breed dogs [59], since pancreatitis will disrupt pancreatic trypsin production. By analogy, fatal type C *C*. *perfringens* enteritis has commonly been associated with trypsin inhibition by foods such as cassava, or by trypsin inhibitory factors in colostrum, with the consequence that CPB toxin produced in the small intestine is not destroyed by the proteolytic action of trypsin but rather initiates intestinal necrosis and cascading clostridial disease [1]. Although there was a highly significant association between the presence of *netF* and canine hemorrhagic gastroenteritis compared to undifferentiated canine enteritis, we only obtained *netF*-positive isolates from about 75% of canine hemorrhagic gastroenteritis cases; failure to isolate *netF* from all cases might relate to loss of the pCP718netF plasmid, to isolation of non-toxigenic *C*. *perfringens*, to the presence of other etiologic agents, or for other reasons.

Enterocolitis in neonatal foals associated with types A and C *C*. *perfringens* is associated with high mortality [60], but the role of type A isolates has not been defined. This study identified the highly significant association of type A strains producing the novel pore-forming toxin NetF with this disease, and opens the way for control based on immunoprophylaxis or for measures based on future understanding of the epidemiologic basis of the disease [24,60]. Most foals with *C*. *perfringens*-associated enterocolitis have been younger than three days of age [25], also thus supporting a role for the trypsin-inhibitory action of colostrum [61] in interfering with the breakdown of *C*. *perfringens* toxins as an important feature of its pathogenesis. Failure to identify *netF* in all cases of type A *C*. *perfringens*-associated neonatal foal necrotizing enteritis might have explanations similar to those suggested for failure to isolate these bacteria from all cases of canine hemorrhagic gastroenteritis.

## Materials and Methods

### Bacterial Strains, Plasmids and Growth Media

Bacterial strains and plasmids used are described in Tables 4 and 7, respectively**.** *C*. *perfringens* strains were grown overnight at 37°C under anaerobic conditions (80% N_2_, 10% H_2_, 10% CO_2_) on either TPG medium (5% Tryptone [Becton, Dickinson and Company, Sparks, MD], 0.5% proteose peptone [Fisher Scientific, ON], 0.4% glucose [Fisher Scientific], and 0.1% thioglycolic acid [Sigma-Aldrich, St. Louis, MO]) or Brain Heart Infusion (BHI) agar (Becton, Dickinson and Company). All *C*. *perfringens* isolates were also cultivated in blood agar (Trypticase Soy Agar [Fisher Scientific] with 5% sheep blood) plates aerobically to confirm purity. *E*. *coli* strains DH5α (Stratagene, La Jolla, CA) was used as the host for plasmid construction, *E*. *coli* BL21-Star (DE3) pLysS (Invitrogen, Carlsbad, CA) was used for the overexpression of histidine-tagged fusion proteins, and *E*. *coli* CA434 for conjugation assays (Table 4). *E*. *coli* strains were grown on Luria-Bertani (LB) broth or agar plates (Becton, Dickinson and Company) overnight at 37°C. All strains were stored at -70°C in lyophilizing medium (25 g powdered skim milk [Fisher Scientific], 18.75 g glucose, 25 g sucrose [Sigma-Aldrich], 2.5 g bovine albumin [Sigma-Aldrich] in 250 ml distilled water).

**Table 7:** Plasmids used in this study.

Plasmid	Relevant characteristics	Source
pJIR750	*E*.* coli—C*.* perfringens* shuttle vector, Cm^R^	J.I.Rood, Monash University
pNetF07	pJIR750Cm^R^ containing *netF* gene and 250 bp of its upstream region	This study
pMTL007	Inducible clostridial expression vector for expression of ClosTron, containing Erm RAM, ColE1, pCB102, Cm^R^	[62]
pMTLnetE	pMTL007 containing intron retargeted to *C*. *perfringens netE* (sense insertion at 883–884 bp)	This study
pMTLnetF	pMTL007 containing intron retargeted to *C*. *perfringens netF* (antisense insertion at 152–153 bp)	This study
pMTLnetG	pMTL007 containing intron retargeted to *C*. *perfringens netG* (antisense insertion at 583–584 bp)	This study
pET-28a	*E*. *coli* expression vector. P_T7_, Kan^R^, *ori* pBR322, *ori* f1, *lac*I, T7, Tag N-terminal 6xHis, C-terminal 6xHis	(Novagen, Gibbstown, NJ)
pET28::*netE*	pET28a containing the DNA fragment amplified by PCR using primers netE-F-*Eco*RI and netE-R- *Hin*dIII	This study
pET28::*netF*	pET28a containing the DNA fragment amplified by PCR using primers netF-F- *Eco*RI and netF-R-*Xho*I	This study
pET28::*netG*	pET28a containing the DNA fragment amplified by PCR using primers netG-F-*Bam*HI and netG-R- *Xho*I	This study
pET43.1a	*E*. *coli* expression vector. P_T7_, Amp^R^, *ori* pBR322, *ori* f1, *lac*I, T7, Tag N-terminal Nus, C-terminal 6xHis	(Novagen, Gibbstown, NJ)
pET43::*netE*	pET43.1a containing the DNA fragment amplified by PCR using primers netE-F- *Eco*RI and netE-R- *Hin*dIII	This study
pET43::*netF*	pET43.1a containing the DNA fragment amplified by PCR using primers netF-F- *Eco*RI and netF-R-*Xho*I	This study
pET43::*netG*	pET43.1a containing the DNA fragment amplified by PCR using primers netG-F-*Bam*HI and netG-R- *Xho*I	This study

### Identification of Necrotizing Toxin Genes

The identification of necrotizing toxin genes *netE*, *netF* and *netG* was made possible by the sequencing of a canine strain of *C*. *perfringens* (JFP718) carried out by the McGill University and Genome Quebec Innovation Centre (Montreal, QC) (data not shown). The pseudochromosome, plasmid fragments, and unplaced contigs were automatically annotated by Rapid Annotation using Subsystem Technology (RAST). BLASTN and BLASTX analyzes were performed to compare the established sequences to known *C*. *perfringens* sequences in the NCBI database.

### Genomic and Plasmid DNA Isolation

A 1 ml aliquot of TPG medium cultured at 37°C overnight was spun down at 13,500 x *g* and DNA of the samples were extracted using InstaGene Matrix (Bio-Rad Laboratories, Mississauga, ON) following the manufacturer’s directions**.** Plasmid DNA was purified using midi-Qiagen columns (Qiagen, Mississauga, ON) following the manufacturer’s instructions.

### PCR Amplifications for Toxin Genes

Detection of various toxin genes was carried out by PCR amplification of each *C*. *perfringens* isolate. The presence of the *netE*, *netF* and *netG* toxin genes was detected by PCR amplification, using primers designed from the *C*. *perfringens* JFP718 sequences. PCR-based toxin gene identification of *cpa*, *cpb2* and *cpe* was also done. Primers used are described in S2 Table. Amplifications were performed in a 25 μl total volume containing the following: 5 μl of template DNA; 1X PCR buffer with Mg^2+^ (New England BioLabs, Pickering, ON); 0.2 mM deoxynucleoside triphosphate mixture; 2.5 units of TaqDNA polymerase (New England BioLabs); and 200 nM of each primer. The PCR program for *netF* was: 94°C for 3 min, 30 cycles of 94°C for 30 sec, 45°C for 30 sec, extension at 72°C for 1 min, and finally, 72°C for 5 min. The PCR programs for *netE* and *netG* were similar with the exception of the annealing temperature, 48°C for 30 sec/cycle. PCR product sizes were determined by agarose gel electrophoresis and visualized by ethidium bromide staining and photographed under UV light.

#### Prevalence of *netE*, *netF* and *netG* Genes in *C*. *perfringens* Isolates from Different Animal Species

*C*. *perfringens* type A isolates examined were from a collection of equine (n = 84), canine (n = 92), bovine (n = 47), caprine (n = 24) and ovine (n = 28) origin. Some isolates were from cases of diarrheal disease in animals presented to the Animal Health Laboratory, University of Guelph, some of which had detailed pathological diagnostic descriptions that included cases of fatal enteritis. Other isolates were from diarrheic horses (Dr. J.S. Weese, University of Guelph) or calves (Dr. K. Leslie, University of Guelph). In addition, some isolates were from fatal necrotizing enteritis in foals or fatal canine hemorrhagic enteritis (Dr. T. Besser, Washington State University, Pullman, WA) or originated from undifferentiated diarrheal illness in dogs (Dr. R. J. Carman, TechLabs, Blacksburg, VA; Dr. V. Perreten, Institute of Veterinary Bacteriology, University of Bern, Switzerland). Positive identifications of *C*. *perfringens* were based on the presence of the characteristic double zone of hemolysis and colonial morphology as well as the presence of the alpha-toxin gene *cpa* by PCR.

### Cytotoxicity Assay and Cell lines

Culture supernatants of equine and canine *netE*, *netF*, and *netG* positive *C*. *perfringens* isolates were evaluated for cytotoxicity on several cell lines. Equine Ovarian cell line, EO (Dr. E. Nagy, University of Guelph; [63]); Madin Darby Bovine Kidney cell line, MDBK (ATCC, CCL-22); Madin Darby Canine Kidney cell line, MDCK (ATCC, CCL-34); Porcine Kidney cell line, PK15 (ATCC, CCL-33); Rat Fischer Fibroblast cell line, 208F (Life Technologies, ON); Mouse Embryo Fibroblast cell line, NIH 3T3 (ATCC, CRL- 1658); African green monkey kidney cell line, Vero (ATCC, CCL-81); and human colon epithelial cell line, CaCo-2 (ATCC, HTB-37) were grown in EMEM complete media (EMEM 450 ml, 10% fetal calf serum [VWR International, Radnor, PA], 2 mM L-glutamine [Sigma-Aldrich]). The A72, a cell line derived from canine fibroblasts, was maintained in Dulbecco's Modified Eagle Medium (DMEM) supplemented with 10% fetal calf serum [VWR International], 2 mM L-glutamine [Sigma-Aldrich]. The primary chicken hepatocellular carcinoma epithelial cell line, LMH (ATCC, CRL-2117) was grown in Waymouth's complete media (90% Waymouth's MB 752/1 [Invitrogen, ON], 10% fetal calf serum). All cells were incubated at 37°C in an atmosphere of humidified 5% CO_2_. To test for cytotoxicity, cell lines were cultured to almost 100% confluence in 96-well plates (Corning Inc., Corning, NY) grown in their respective growth medium in 5% CO_2_ at 37°C.

For bacterial cytotoxin testing, bacterial supernatants from TPG broth cultures with an OD_600_ of 0.6–0.8 were filtered through a 0.22 μm filter (Fisher Scientific). Subsequently, 100 μl of sterile bacterial culture supernatant was added to the wells, in a 2-fold dilution series up to 1:1024 (v/v) in duplicate for each strain tested. Cytotoxicity was evaluated microscopically over 8 h as described in detail in Mehdizadeh Gohari *et al*. [64]. *C*. *perfringens* strains NCTC3110 (*cpb*-positive) and CW504 (*cpa*-positive) were used as positive and negative controls, respectively. The titer was the final dilution that showed >2+ cytotoxicity.

### Mutation of *netE*, *netF*, and *netG* Toxin Genes and Conjugation

The generation of *C*. *perfringens* mutants was conducted as described in Heap *et al*. [62]. The ClosTron intron targeting and design tool (http://clostron.com) identified possible intron target sites. The insertion sites at the positions 883/884bp in the *netE* open-reading frame (ORF), at positions 152/153bp in the *netF* ORF, and positions 583/584 bp in the *netG* ORF were preferentially chosen to generate ClosTron-intron modifications, which were obtained by PCR from primers (IBS, EBS2, EBS1 and EBS universal) designed by the ClosTron website (S2 Table). The 350 bp PCR products and pMTL007 ClosTron-shuttle vector were digested with *Hin*dIII and *Bsr*GI, ligated and then transformed by heat shock into *E*. *coli* DH5α. Recombinant plasmids were isolated and sequenced in order to verify sequences of the retargeted intron specific for *netE*, *netF* and *netG* insertions. The recombinants pMTLnetF, pMTLnetE and pMTLnetG containing the modified *netE*, *netF* and *netG* intron were then electroporated into electrocompetent *E*. *coli* CA434 for conjugation experiments. Conjugation was conducted as described in Heap *et al*. [65]. Plasmid transfer experiments were carried out with *E*. *coli* CA434 carrying recombinant plasmids pMTLnetE, pMTLnetF, and pMTLnetG, respectively. Overnight BHI cultures of the resultant *E*. *coli* CA434 were used as donor strains and the equine-source *C*. *perfringens* JFP838 strain was used as recipient. Donor and recipient cells were mixed at a ratio of 3:1 and a total of 200 μl of both cultures were spread onto BHI agar without antibiotics and incubated anaerobically at 37°C overnight. Subsequently, the bacterial growth was removed and resuspended in 1 ml of PBS. Transconjugants were selected on BHI agar plates containing cycloserine (250 μg/ml) and thiamphenicol (15 μg/ml). Transconjugants screening were performed as described in Parreira *et al*. [4]. Transconjugants were confirmed by PCR amplifications of specific genes (*netE*, *netF* and *netG*) (S2 Table).

#### Complementation of *C*. *perfringens* JFP838::*netF*

For complementation assays, the plasmid pJIR750 was used to construct the recombinant pNetF07 which contains the entire *netF* gene together with the 250 bp upstream region. *C*. *perfringens* JFP838::*netF* mutant was complemented with pNetF1A by the same conjugation method described above with *E*. *coli* CA434 as donor strain, using erythromycin and thiamphenicol to select transconjugants. The recombinant plasmid pNetF was also introduced into *C*. *perfringens* JIR325in order to analyze NetF expression and cytotoxicity.

### Construction and Purification of Recombinant Toxins NetE, NetF and NetG using pET-28a

The chromosomal DNA of *C*. *perfringens* strain JFP728 was used as a template in PCR reactions. PCR reactions were performed with a Platinum PCR SuperMix high-fidelity kit (Invitrogen) and specific primers are described in S2 Table. The PCR amplified *netE*, *netF* and *netG* genes were cloned into the *Eco*RI—*Hin*dIII, *Eco*RI—*Xho*I, and *Bam*HI—*Xho*I sites of vector pET-28a vector (Novagen, Gibbstown, NJ) to generate proteins fused with histidine residues (6-His), then transformed into *E*. *coli* DH5α. The nucleotide sequences of the cloned PCR products were verified by sequencing. The resulting plasmids (pET28::*netE*, pET28::*netF*, pET28::*netG*) were introduced into *E*. *coli* BL21-Star (DE3) pLysS. The recombinant *E*. *coli* BL21 strains harboring recombinant plasmids were grown in LB medium with kanamycin (50 μg/ml) and chloramphenicol (34 μg/ml) at 37°C until the absorbance at 600 nm reached 0.5, and then induced by adding 1 mM isopropyl-b-D-thiogalactopyranoside (IPTG) at 37°C for 4 h. Purification of recombinant His-tagged NetE, NetF and NetG proteins from *E*. *coli* BL21 was performed under denaturing conditions according to the manufacturer’s instructions (Qiagen). Briefly, for purification, the cell pellet was resuspended in 5 ml of lysis buffer (100 mM NaH_2_PO_4_, 10 mM Tris-Cl, 8M Urea, pH 8.0). The mixture was stirred for 60 min at room temperature. The lysate was centrifuged at 10,000xg for 30 min at room temperature to harvest the cellular debris. One ml of the 50% affinity chromatography on nickel-nitrilotriacetic acid agarose (Ni-NTA) was added to cleared lysate and mixed gently on a shaker for 60 min at 4°C. The column was washed twice with 4 ml of lysis buffer- pH 6.3, followed by 4 times with 1 ml lysis buffer- pH 5.9, and rNetE, rNetF and rNetG eluted with 4 times lysis buffer- pH 4.5. The final products were characterized by SDS-PAGE analysis.

### Construction and Purification of Recombinant Toxins NetE, NetF and NetG using pET43.1a

The PCR amplified *netF* gene was cloned into the *Eco*RI and *Xho*I sites of vector pET43.1a (Novagen), whereas PCR products of *netE* and *netG* genes were cloned into the *Eco*RI—*Hin*dIII and *Bam*HI—*Xho*I sites of vector pET43.1a. Transformation into expression strain (*E*. *coli* BL21) and induction was done as described above. Purification of rNetE-NusA, rNetF-NusA and rNetG-NusA was done by Ni-NTA agarose under native conditions following the manufacturer’s instructions (Qiagen). The resulting protein expressions and solubility levels were evaluated by SDS gel electrophoresis.

#### Horse Immunization using rNet-NusA Proteins

The recombinant fusion proteins were used for polyclonal antibody production in 7 adult horses (3 horses for rNetF-NusA and 2 horses each for rNetE-NusA or rNetG-NusA). A 1:4 ratio of aluminium hydroxide gel and sterile antigen was mixed together using 2.0 mg of antigen in 2 ml PBS, for a total of 2.5 ml per horse. For the primary immunization 1.0 mg of recombinant fusion protein and for the 2 subsequent immunizations 2.0 mg of fusion protein were used. Horses were immunized intramuscularly at day 0, 14, and 28, and bled at these times as well as on day 35 and 42 after initial immunization. Horses were examined for 4 days after each immunization and body reactions to injected antigen were recorded.

#### Cytotoxin Neutralization by Antibodies to rNetF, rNetE and rNetG

The neutralization of cytotoxicity by equine polyclonal antibody produced against each of the rNet-NusA proteins was performed with the EO cell line. An overnight culture supernatant of a *netE*, *netF* and *netG*-positive strain (JFP728) was prepared as described above. The supernatant was diluted in EMEM medium; the dilution of supernatant used for determination of neutralization titers was 128. Subsequently, serial 2-fold dilutions of sera up to 1:102,400 were made in a new 96-well plate (100 μl/well). Diluted antibodies were transferred into the diluted toxin plate and manually homogenized for 30 sec, then incubated for 2 h at 37°C. After incubation, 100 μl of the toxin-antibody dilution series was added into a plate with confluent EO cells which was incubated in a humidified environment of 5% CO_2_ at 37°C for 8 h. The neutralizing antibody titer was that showing an inhibition of 2+ or greater [66].

#### Enzyme-Linked Immunosorbent Assay (ELISA)

rNetE, rNetF and rNetG separated by SDS-PAGE were recovered by electro-elution using the Bio-Rad model 422 electro-eluter according to manufacturer’s protocol. Briefly, recombinant proteins were electrophoresed on a 12% polyacrylamide gel and the zone with rNet protein was cut from the gel, macerated into small pieces and then placed in electro-elution tubes containing electrophoretic buffer (25 mM Tris base, 192 mM Glycine and 0.1% SDS). Transferred recombinant proteins underwent dialysis to remove SDS.

For ELISA, 96-well plates (MaxiSorp, Nunc, Roskilde, Denmark) were coated with 0.5 μg /well of individual electro-eluted rNet proteins in carbonate-bicarbonate buffer pH 9.6 for 1 h at 37°C followed by overnight incubation at 4°C [67]. Briefly, plates were washed twice with buffer (phosphate-buffered saline pH 7.4 (PBS), 0.05% Tween 20) and once with PBS, and the coated plates were blocked using blocking buffer (PBS, 0.05% Tween 20, 0.5% fish skin gelatin [Norland HiPure Liquid Gelatin, Norland Products Inc, Cranbury, NJ]) for 2 h at 37°C. After washing 3 times with wash buffer, 100 μl/well of horse polyclonal serum (2-fold serial dilutions up to 1:409600) were added to the plate in duplicate. Washing buffer with no polyclonal antibodies was used as a negative control. After incubation at room temperature for 2 h, followed by 3 times washings with washing buffer, 100 μl/well of enzyme-labeled detecting goat anti-horse antibody (Jackson ImmunoResearch Laboratories Inc., West Grove, PA) (diluted 1:5000 in wash buffer) was applied, and the plate was incubated for another 1 h at room temperature. The plate was then washed 3 times with wash buffer and 100 μl/well of the chromogenic substrate 2,2'-azino-di-[3-ethylbenzthiazoline sulfonate] diammonium salt (ABTS) (Roche Applied Science) was added. The reaction was stopped after 30 min to 1 h of further incubation at room temperature using 0.5% SDS (50 μl/well) and the OD measured at 405 nm in an ELISA reader (BioTek Instruments Inc., Power Wave XS, Winooski, VT).

#### Western Blot Analysis

Immunoblotting was used for three main reasons. Firstly, to assess the specificity of horse polyclonal serum prepared against rNet proteins. For this purpose, 2 type B *C*. *perfringens* (NCTC3110, NCTC7368), 2 type C *C*. *perfringens* (ATCC3628, NCTC3181), and 2 *netB*-positive *C*. *perfringens* strains (CP1, CP4) were tested. Secondly, to confirm the mutation of three *net* genes and complementation of *netF* gene, as well as expression of all three native Net proteins in wild-type strain. For this, 3 mutant strains (JFP838E-05, JFP838F-05 and JFP838G-07), complemented *netF* strain (VN-22C), and wild-type strain (JFP838) were tested using horse polyclonal serum prepared against rNet proteins. Thirdly, to indicate the absence of expression of the *cpe* gene under the growth conditions used in this study. For this purpose, wild-type strain (JFP383) was tested using sheep polyclonal rCPE antibody (Dr. Michel Popoff, Institut Pasteur, France, and recombinant CPE toxin (TechLab) was used as a positive control.

Broth culture supernatants were mixed with a 1:1 ratio of Laemmli Sample Buffer (Bio-Rad) and separated by SDS-PAGE in 12% acrylamide gel. Subsequently, proteins were transferred onto a nitrocellulose 0.45 μm membranes (BioTrace NT, Gelman Laboratory, Laurent, QC) for 60 min at constant power supply of 95 V. Membranes were then placed in blocking buffer (PBS, 0.05% Tween 20, 0.5% fish skin gelatin) at 4°C overnight, followed by incubation with serum from horses immunized with 1 of the 3 rNet-NusA proteins at 1:2000 dilution, for 90 min at room temperature. After washing 3 times, the membranes were incubated with alkaline phosphatase-conjugated goat anti-horse IgG (Jackson ImmunoResearch Laboratories) at 1:5000 dilution. Specific protein bands were visualized using the alkaline phosphatase conjugate substrate kit (Bio-Rad) [68]. BLUeye Prestained Protein Ladder (FroggaBio, ON) was used in Western blot analysis. For the third experiment, the same condition was employed. The only difference was that sheep polyclonal serum against rCPE and alkaline phosphatase-conjugated goat anti-sheep IgG (Jackson ImmunoResearch Laboratories) were used in this experiment.

### Pulse Field Gel Electrophoresis (PFGE)

PFGE was performed to analyze the presence of plasmids in 12 *C*. *perfringens* isolates (6 canine isolates and 6 equine isolates), as described by Parreira *et al*. [4], and on chromosomal DNA to examine the clonality of 35 equine (16 *netF*+, 19 *netF*-) and 35 canine (16 *netF*+, 19 *netF*-) *C*. *perfringens* isolates, using the method described by Chalmers *et al*. [50]. NotI digestion was used to linearize plasmids since this cuts large Tcp-positive conjugative *C*. *perfringens* plasmids only once [4, 51]. Thirty-two isolates were selected on the basis of the presence of the *netF* gene, and thirty-eight isolates were randomly selected from fecal isolates from diarrheal or healthy animals that were negative for the *netF* gene. Electrophoresis was performed in a 1% PFGE-certified gel and separated with the CHEF-III PFGE system (Bio-Rad) in 0.56 Tris-borate-EDTA buffer supplemented with 200 μM thiourea (Fisher Scientific) at 14°C at 6 V for 19 h with a ramped pulsed time of 1 to 12 sec (plasmid protocol) and 4 to 38 sec (clonality protocol). As a size guide, *C*. *perfringens* strain 33 was electrophoresed on each gel, with markers being used in lanes 1, 2, 7 and 15. Gels were stained in ethidium bromide and visualized by UV light. Mid-Range II PFG markers (New England Biolabs) were used as the molecular DNA ladder.

Band matching was performed using a 0.8% position tolerance; cluster analysis was completed utilizing the Dice similarity coefficient and unweighted pair group method with arithmetic mean. The isolate host species (equine or canine), laboratory numeric code, and presence of *netF* (+ or-) were included. Genotypes were designated through application of the Tenover criteria [69] with 60% band pattern similarity equating to a 7-band difference. These genotypes were labelled alphabetically from top to bottom, with a subscript representing the percent relatedness of the banding pattern. Analysis of the PFGE gels was completed utilizing BioNumerics software version 7.1 (Applied Maths, Austin, TX).

#### Preparation of DIG Probes and Plasmid PFGE for Southern Blotting

DNA probes for all plasmid PFGE Southern blot steps were labelled by PCR amplification in the presence of digoxigenin-11-dUTP (DIG; Roche Applied Science) according to the manufacturer’s recommendation. DNA probes were amplified from *C*. *perfringens* strain JFP718. DNA probes for *netE* and *cpe* genes were prepared with specific primers (S2 Table). DNA from PFGE gels was transferred to nylon membranes (Roche Applied Science, Mannheim, Germany). DNA hybridizations and detection were performed by using the DIG labelling and CSPD substrate according to the manufacturer’s recommendation (Roche Applied Science). For Southern blot hybridizations, nylon membranes were prehybridized for at least 2 h at 42^°^C in hybridization solution without labelled probe and then hybridized separately at 42^°^C with specific DNA probes for 16 h. The membranes were washed at 68^°^C under high-stringency conditions. For each different DIG labelled probe, the membrane was first stripped with 0.2 N NaOH and 0.1% sodium dodecyl sulfate, incubated with prehybridization solution, and then reprobed.

### Nucleotide Sequence Accession Numbers

The *net* toxin sequences were assigned GenBank accession numbers KJ606985 for *netE*, KJ606986 for *netF* and KJ606987 for *netG*. The incomplete plasmid sequences are available in GenBank as KP739975 (pCP718netF [scaffold 00006]) and KP739976 (pCP718cpe [scaffold 00012]).

### Statistical Analyses

Fisher’s exact test was used to make Odds Ratio estimates about the association of *netF* with canine hemorrhagic gastroenteritis or foal necrotizing enteritis, since the test uses the hypergeometric distribution that allows us to make exact statements. Conditional means likelihood estimates (CMLE) of Odd Ratios were obtained, with 95% confidence intervals, except where one of the cell counts was 0, in which case mean unbiased estimates (MUE) were determined [70].

### Ethics Statement

Horse immunization studies were approved by the University of Guelph Animal Care Committee (Animal Utilization Protocol approval number 1818) in accordance with the guidelines of the Canadian Council on Animal Care.

## Supporting Information

S1 Fig Sequence alignment of toxins from *Clostridium perfringens*. Residue numbers for individual proteins are given on the top of the sequences. Filled- in boxes represent identical residues, whereas outlined boxes represent similar residues. The secondary structure is shown on top based on their respective crystallographic structures (β = beta-strand, η = 3_10_ helix, α = alpha-helix, strict β-turns = TT and strict α-turns = TTT). The alignment was performed using ClustalW of the toxins Panton-Valentine leukocidin S of *Staphylococcus aureus* (AHC29058), putative CctA of *C*. *chauvoei* (WP_021874975), alpha-hemolysin of *C*. *botulinum* (YP_004394739.1), NetE (KJ606985), NetF (KJ606986), NetG (KJ606987) of *C*. *perfringens*, NetB of *C*. *perfringens* (EU143239) and Delta toxin of *C*. *perfringens* (EU652406) and Beta-toxin of *C*. *perfringens* (CAA58246.1). The figure was created using the ESPript 2.2.program.
(TIFF)

S2 Fig Southern blot (SB) analysis of genomic DNA from mutants (JFP838E-05, JFP838F-05 and JFP838G-07) and wild type strain JFP838 digested with *Hind*III using intron probe. SB was performed using DIG system (Roche Applied Science) for labelling and detection according to the manufacturer’s recommendation. PCR probe was amplified using probe Intron primer pairs (S2 Table) designed based on the group II intron sequence, Lane 1: JFP838E-05, Lane 2:JFP838F-05, Lane 3:JFP838G-07, Lane 4: wild-type JFP838, Lane 5: DNA DIG-ladder VII (Roche).
(TIF)

S3 Fig ClosTron-based mutation for inactivation of genes *netE*, *netF* and *netG*. PCR amplifications of genomic DNA of mutants *netE*, *netF* and *netG* (A) using specific primer pairs (netE-F/R, netF-F/R, netG-F/R described on S2 Table) Lane M: 1 kb ladder (NEB), Lane 1: JFP838E-05, Lane 3: JFP838F-05, Lane 5: JFP838G-07 and Lanes 2,4,6: wild-type JFP838 and (B) using target primers (netE-F, netF-R and netG-R) in combination with EBS-Universal primer. Lane M: 100 bp ladder (NEB), Lane 1: JFP838E-05, Lane 3: JFP838F-05, Lane 5: JFP838G-07 and Lanes 2,4,6: wild-type JFP838.
(TIFF)

S4 Fig Immunoblots showing production of NetF by the complemented mutant, NetE and NetG production, and failure to produce CPE under the conditions used. Fig. A: Western blot using horse polyclonal Ab against rNetF. Lane 1: Culture supernatant of wild-type *netF*-positive strain (JFP838); Lane 2: Culture supernatant of mutant strain, showing absence of NetF; Lane 3: Culture supernatant of complementing strain, showing production of NetF. Fig. B: Western blot using horse polyclonal Ab against rNetG. Lane 1: Culture supernatant of *netG* mutant strain, showing absence of NetG; Lane 2: Culture supernatant of wild-type of *netG*; positive strain. Fig. C: Western blot using horse polyclonal Ab against rNetE. Lane 1: Culture supernatants of *netE* mutant strain, showing absence of NetE; Lane 2: Culture supernatant of wild-type *netE* positive strain. Fig. D: Immunoblot using sheep polyclonal Ab against CPE showing the lack of expression of CPE under the growth condition used in this study. Lane 1: Purified rCPE (positive control); Lane 2: Culture supernatant of a canine *netF-* and *cpe*-positive strain (JFP718).
(TIF)

S1 Table Presence of VirR box upstream Net genes. (DOCX)

S2 Table Primers used in this study. (DOCX)
