# Supplementary figures and images for: A Novel Pore-Forming Toxin in Type A Clostridium perfringens Is Associated with Both Fatal Canine Hemorrhagic Gastroenteritis and Fatal Foal Necrotizing Enterocolitis
Source: PLoS One. 2015 Apr 8;10(4):e0122684. doi: 10.1371/journal.pone.0122684 (PMC4390311; doi:10.1371/journal.pone.0122684)

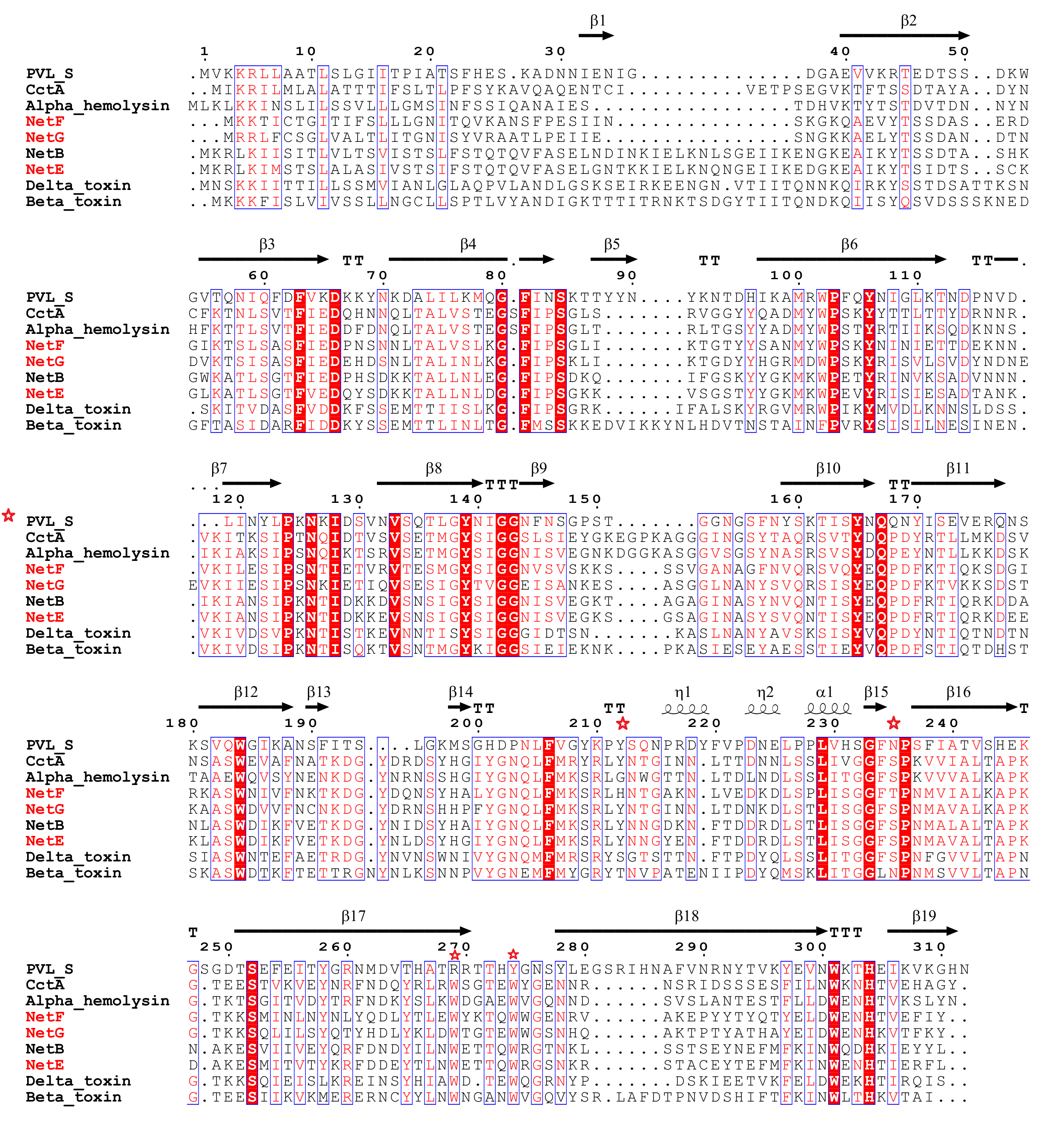

Supplement: S1 Fig — Residue numbers for individual proteins are given on the top of the sequences. Filled- in boxes represent identical residues, whereas outlined boxes represent similar residues. The secondary structure is shown on top based on their respective crystallographic structures (β = beta-strand, η = 310 helix, α = alpha-helix, strict β-turns = TT and strict α-turns = TTT). The alignment was performed using ClustalW of the toxins Panton-Valentine leukocidin S of Staphylococcus aureus (AHC29058), putative CctA of C. chauvoei (WP_021874975), alpha-hemolysin of C. botulinum (YP_004394739.1), NetE (KJ606985), NetF (KJ606986), NetG (KJ606987) of C. perfringens, NetB of C. perfringens (EU143239) and Delta toxin of C. perfringens (EU652406) and Beta-toxin of C. perfringens (CAA58246.1). The figure was created using the ESPript 2.2.program. (TIFF) [file pone.0122684.s001.TIFF]

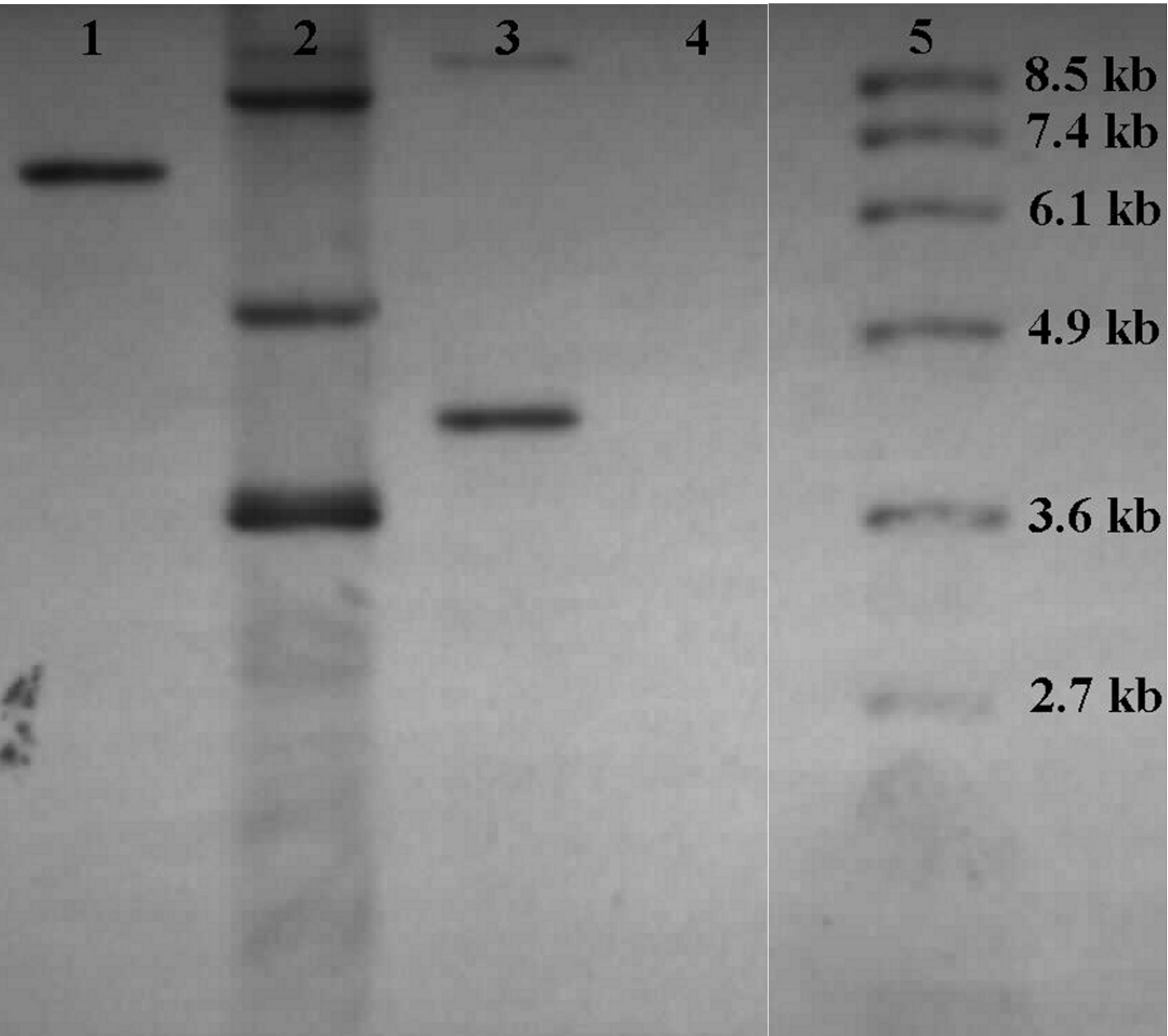

Supplement: S2 Fig — SB was performed using DIG system (Roche Applied Science) for labelling and detection according to the manufacturer’s recommendation. PCR probe was amplified using probe Intron primer pairs (S2 Table) designed based on the group II intron sequence, Lane 1: JFP838E-05, Lane 2:JFP838F-05, Lane 3:JFP838G-07, Lane 4: wild-type JFP838, Lane 5: DNA DIG-ladder VII (Roche). (TIF) [file pone.0122684.s002.TIF]

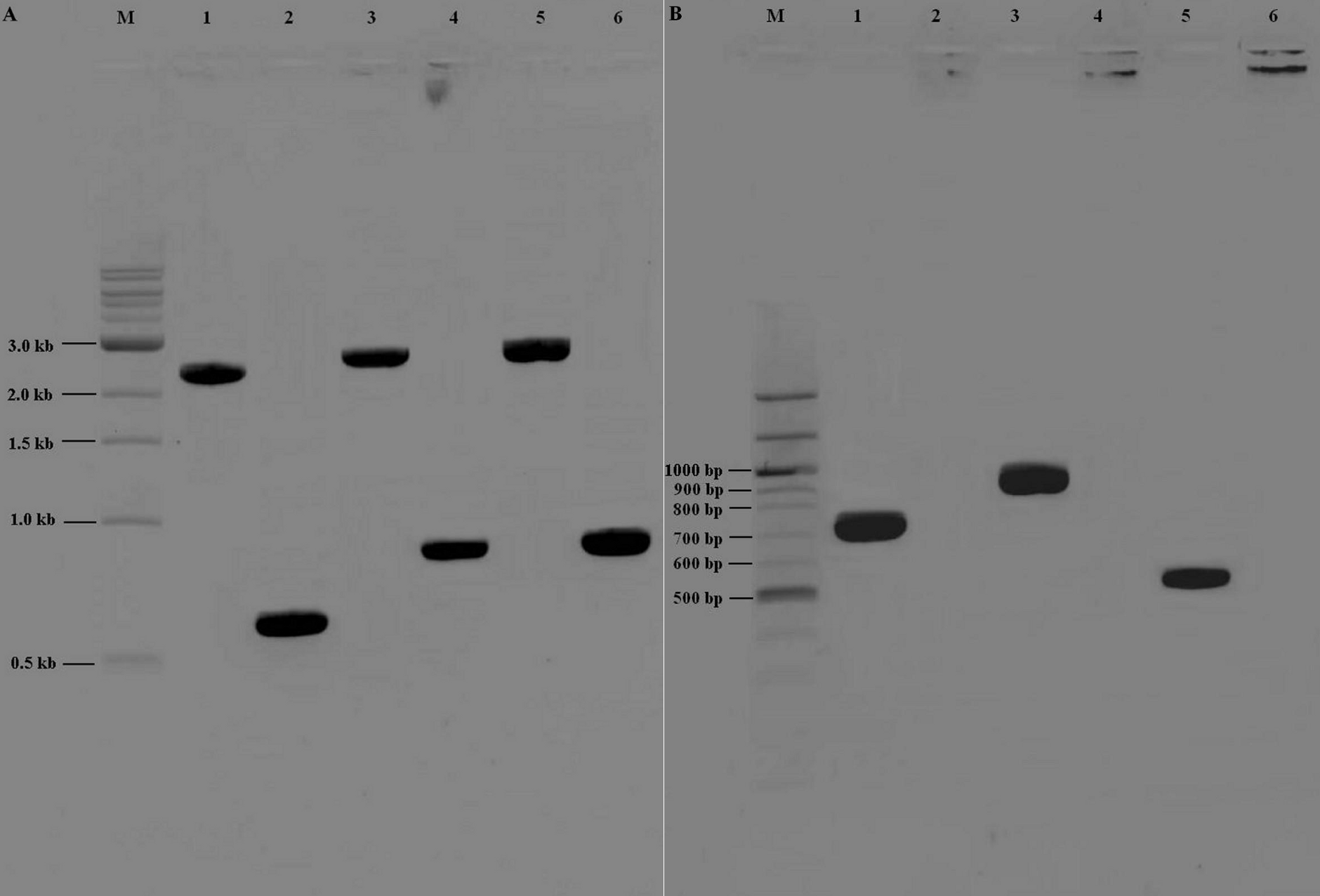

Supplement: S3 Fig — PCR amplifications of genomic DNA of mutants netE, netF and netG (A) using specific primer pairs (netE-F/R, netF-F/R, netG-F/R described on S2 Table) Lane M: 1 kb ladder (NEB), Lane 1: JFP838E-05, Lane 3: JFP838F-05, Lane 5: JFP838G-07 and Lanes 2,4,6: wild-type JFP838 and (B) using target primers (netE-F, netF-R and netG-R) in combination with EBS-Universal primer. Lane M: 100 bp ladder (NEB), Lane 1: JFP838E-05, Lane 3: JFP838F-05, Lane 5: JFP838G-07 and Lanes 2,4,6: wild-type JFP838. (TIFF) [file pone.0122684.s003.TIFF]

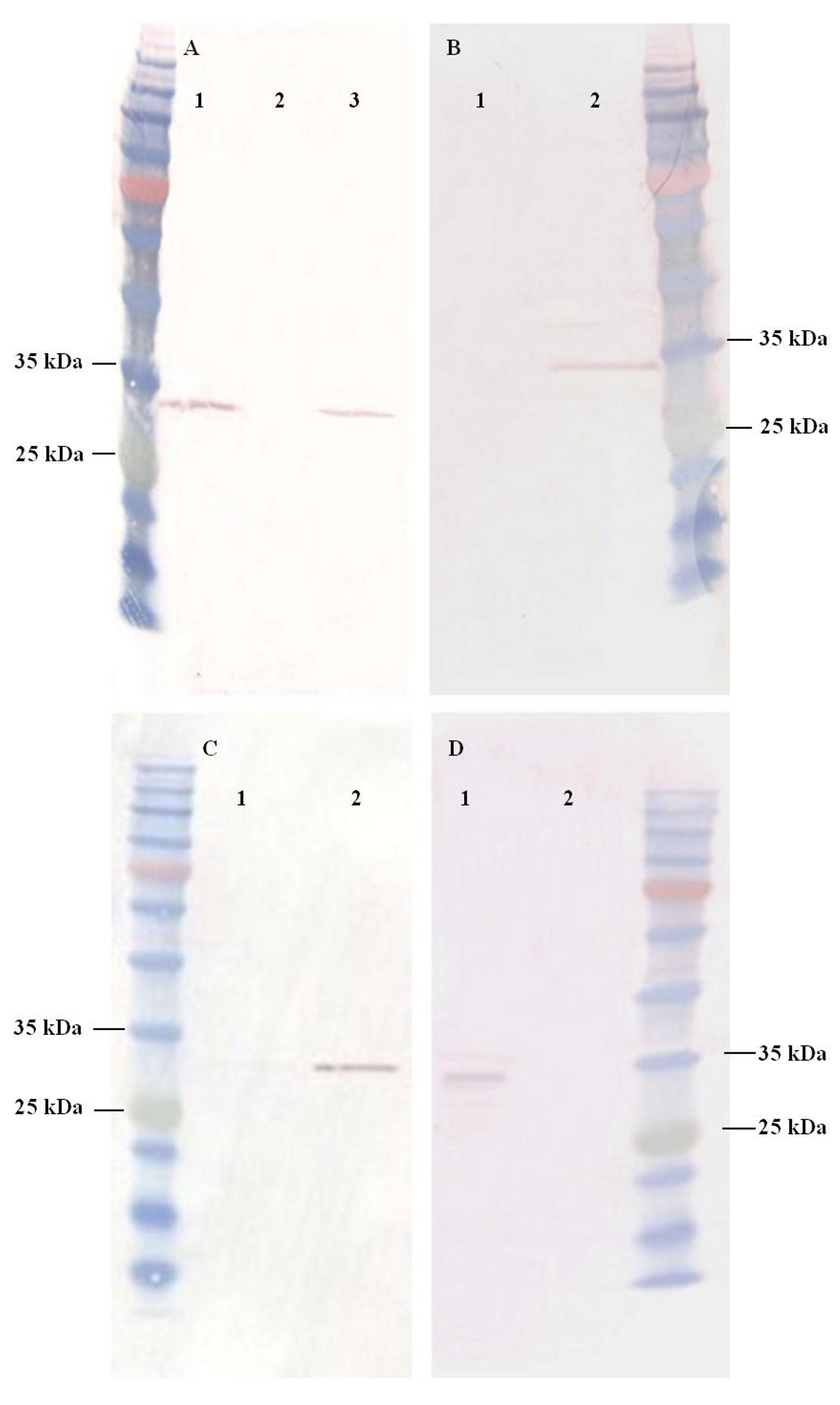

Supplement: S4 Fig — Fig. A: Western blot using horse polyclonal Ab against rNetF. Lane 1: Culture supernatant of wild-type netF-positive strain (JFP838); Lane 2: Culture supernatant of mutant strain, showing absence of NetF; Lane 3: Culture supernatant of complementing strain, showing production of NetF. Fig. B: Western blot using horse polyclonal Ab against rNetG. Lane 1: Culture supernatant of netG mutant strain, showing absence of NetG; Lane 2: Culture supernatant of wild-type of netG; positive strain. Fig. C: Western blot using horse polyclonal Ab against rNetE. Lane 1: Culture supernatants of netE mutant strain, showing absence of NetE; Lane 2: Culture supernatant of wild-type netE positive strain. Fig. D: Immunoblot using sheep polyclonal Ab against CPE showing the lack of expression of CPE under the growth condition used in this study. Lane 1: Purified rCPE (positive control); Lane 2: Culture supernatant of a canine netF- and cpe-positive strain (JFP718). (TIF) [file pone.0122684.s004.TIF]
