# Supplementary material for: A Novel Pore-Forming Toxin in Type A Clostridium perfringens Is Associated with Both Fatal Canine Hemorrhagic Gastroenteritis and Fatal Foal Necrotizing Enterocolitis
Source: PLoS One. 2015 Apr 8;10(4):e0122684. doi: 10.1371/journal.pone.0122684 (PMC4390311; doi:10.1371/journal.pone.0122684)
Supplement: S1 Table — (DOCX) [file pone.0122684.s005.docx]

S1Table. Presence of VirR box upstream Net genes.

| **scaffold** | **Gene** | **Orientation** | **Distance (bp)** | **VirR box** |
| --- | --- | --- | --- | --- |
| 00006 | *netE* | - | 300 | cCCAGTTTTACACGAATTTTGACCAGTTATGTA |
| 00006 | *netF* | + | - | - |
| 00012 | *netG* | + | 487 | aCCAGTTATGTATATATTTTGACCAGTTTTACA |
| Consensus VirR box [38] highlighted | | | | cCCAnTTnTncatnannnnTGnCCAGTTntnCAc |
