# Supplementary material for: A Novel Pore-Forming Toxin in Type A Clostridium perfringens Is Associated with Both Fatal Canine Hemorrhagic Gastroenteritis and Fatal Foal Necrotizing Enterocolitis
Source: PLoS One. 2015 Apr 8;10(4):e0122684. doi: 10.1371/journal.pone.0122684 (PMC4390311; doi:10.1371/journal.pone.0122684)
Supplement: S2 Table — (DOCX) [file pone.0122684.s006.docx]

S2 Table. Primers used in this study.

| **Primer names** | **5’-3’** | **PCR size (bp)** | **References** |
| --- | --- | --- | --- |
| **PCR** |  |  |  |
| Ext-netE-F | AATTCAGTATATTCACATGCAG | 1026 | This study |
| Ext-netE-R | CAGTTATACCGATTGTATTAGA |  |  |
| netE-F | TAGAAAACGTTCAATTGTATGG | 601 | This study |
| netE-R | AGAAAGCGCTGATACAGCTAATAAA |  |  |
| netF-F | AACAATATGTACAGGTATAACT | 862 | This study |
| netF-R | TTGATAGGTATAATATGGTTCT |  |  |
| netG-F | TTGTTCAGGATTAGTAGCATTA | 860 | This study |
| netG-R | CATGAGTTGCATAAGTTGGTGT |  |  |
| alpha-toxin-F | GCTAATGTTACTGCCGTTGA | 325 | [64] |
| alpha-toxin-R | CCTCTGATACATCGTGTAAG |  |  |
| beta2-F | ATTATGTTTAGGAATACAGTTA | 741 | [65] |
| beta2-R | CAATACCCTTCACCAAATACTC |  |  |
| enterotoxin-F | GGAGATGGTTGGATATTAGG | 223 |  |
| enterotoxin-R | GGACCAGCAGTTGTAGATA |  |  |
| **Mutation** |  |  |  |
| NetE-IBS | AAAAAAGCTTATAATTATCCTTAAACGTCCAACTGGTGCGCCCAGATAGGGTG | 350 | This study |
| NetE-EBS-1d | CAGATTGTACAAATGTGGTGATAACAGATAAGTCCAACTGCATAACTTACCTTTCTTTGT |  |  |
| NetE-EBS-2 | TGAACGCAAGTTTCTAATTTCGGTTACGTTCCGATAGAGGAAAGTGTCT |  |  |
| NetF-IBS | AAAAAAGCTTATAATTATCCTTAGTCTTCATACCAGTGCGCCCAGATAGGGTG | 350 | This study |
| NetF-EBS-1d | CAGATTGTACAAATGTGGTGATAACAGATAAGTCATACCATCTAACTTACCTTTCTTTGT |  |  |
| NetF-EBS-2 | TGAACGCAAGTTTCTAATTTCGATTAAGACTCGATAGAGGAAAGTGTCT |  |  |
| NetG-IBS | AAAAAAGCTTATAATTATCCTTATCCTACCATAACGTGCGCCCAGATAGGGTG | 350 | This study |
| NetG-EBS-1d | CAGATTGTACAAATGTGGTGATAACAGATAAGTCCATAACCATAACTTACCTTTCTTTGT |  |  |
| NetG-EBS-2 | TGAACGCAAGTTTCTAATTTCGGTTTAGGATCGATAGAGGAAAGTGTCT |  |  |
| EBS-Universal | CGAAATTAGAAACTTGCGTTCAGTAAAC |  |  |
| **Recombinant Protein** |  |  |  |
| RecNetE-F (*Eco*RI) | CCGCGAATTCTCTACTAGTTTAGCTCTTGCAAG | 957 | This study |
| RecNetE-R (*Hin*dIII) | CCGCAAGCTTTAGAAAACGTTCAATTGTATGG |  |  |
| RecNetF-F (*Eco*RI) | CCGCGAATTCAATTCCTTTCCTGAAAGTATTA | 863 | This study |
| RecNetF-R (*Xho*I) | CCGCCTCGAGGTATATAAATTCTACAGTATGA |  |  |
| RecNetG-F (*Bam*HI) | CCGCGGATCCGCTACGTTGCCAGAAATTATTG | 866 | This study |
| RecNetG-R (*Xho*I) | CCGCCTCGAGATATTTAAATGTTACTTTATGG |  |  |
| **SB probes** |  |  |  |
| probeNetE-F | CCTTCAACAGATATATTTCCTCCAA | 419 | This study |
| probeNetE-R | ACACAAACTCAAGTGTTTGCAAGT |  |  |
| probeCpe-F | GGAGATGGTTGGATATTAGG | 300 | This study |
| probeCpe-R | GGACCAGCAGTTGTAGATA |  |  |
| probeIntron-F | TGTAGGAGAACCTATGGGAACGAA | 332 | This study |
| probeIntron-R | TCTTGTAAAAACTTCGTCTATATT |  |  |
